# Genome-wide identification of DnaJ gene family in *Catalpa bungei* and functional analysis of *CbuDnaJ49* in leaf color formation

**DOI:** 10.3389/fpls.2023.1116063

**Published:** 2023-02-23

**Authors:** Yingying Yang, Linjiao Zhao, Junhui Wang, Nan Lu, Wenjun Ma, Jiang Ma, Yu Zhang, Pengyue Fu, Chengcheng Yao, Jiwen Hu, Nan Wang

**Affiliations:** ^1^ State Key Laboratory of Tree Genetics and Breeding, Research Institute of Forestry, Chinese Academy of Forestry, Key Laboratory of Tree Breeding and Cultivation of National Forestry and Grassland Administration, National Innovation Alliance of Catalpa bungei, Beijing, China; ^2^ Biotechnology Research Center of China Three Gorges University, Yichang, China; ^3^ Hekou Yao Autonomous County Forestry and Grassland Bureau, Hekou, China; ^4^ State Key Laboratory of Tree Genetics and Breeding, Northeast Forestry University, Harbin, China

**Keywords:** DnaJ, gene family, function analysis, leaf color, *Catalpa bungei*

## Abstract

DnaJs are the common molecular chaperone proteins with strong structural and functional diversity. In recent years, only several DnaJ family members have been found to be able to regulate leaf color, and it remains to be explored whether there are other potential members that also regulate this character. Here, we identified 88 putative DnaJ proteins from *Catalpa bungei*, and classified them into four types according to their domain. Gene-structure analysis revealed that each member of CbuDnaJ family had same or similar exon-intron structure. Chromosome mapping and collinearity analysis showed that tandem and fragment duplication occurred in the process of evolution. Promoter analyses suggested that *CbuDnaJs* might be involved in a variety of biological processes. The expression levels of DnaJ family members in different color leaves of *Maiyuanjinqiu* were respectively extracted from the differential transcriptome. Among these, *CbuDnaJ49* was the largest differentially expressed gene between the green and yellow sectors. Ectopic overexpression of *CbuDnaJ49* in tobacco showed that the positive transgenic seedlings exhibited albino leaves, and the contents of chlorophyll and carotenoid were significantly reduced compared with those of wild type. The results suggested that *CbuDnaJ49* played an important role in regulating leaf color. This study not only identified a novel gene of DnaJ family members regulating leaf color, but also provided new germplasm for landscaping.

## Introduction

Leaf color variation is a common phenomenon in the plant kingdom. With the development and application of leaf color in landscaping and marker-assisted breeding, people began to notice the importance of leaf color traits (Lee et al., 2021). More and more studies have shown that leaf color is an ideal material for studying chloroplast development, chlorophyll synthesis and photomorphogenesis (Zhang et al., 2021b).

The current research on the mechanism of leaf color mainly focused on chlorophyll metabolism pathway, chloroplast formation or development, and nuclear-cytoplasmic signaling pathway. Firstly, chlorophyll metabolism is a regulatory network affected by a variety of enzymes, and any enzyme or gene mutation could cause leaf color variation (Tripathy and Pattanayak, 2012; Hauenstein et al., 2016; Zhang et al., 2021b). For example, inhibition of *CHLH* reduced the activity of magnesium chelating enzyme and decreased the synthesis rate of chlorophyll in tobacco (Papenbrock et al., 2000). Secondly, the chloroplast is the primary organ responsible for pigment synthesis and photosynthesis. Inhibition of each stage would lead to chloroplast development defects and inhibition of pigment synthesis (Wu et al., 2018). Truncated CCT domain genes could impair chloroplast development in barley, resulting in variegated leaves in the seedlings (Li et al., 2019). Thirdly, it is well known that there exists information exchange between chloroplasts and nuclear genes, and the expression defects of retrograde signals in this pathway could easily lead to leaf variation (Griffin et al., 2020; Unal et al., 2020). The GENOMES UNCOUPLED 4 (GUN4) is a typical retrograde regulatory protein, and it could interact with heme-derived linear tetrapyrroles to stimulate the enzymatic activity of magnesium chelatase (MgCh) (Chan et al., 2010). As mentioned above, most of the current studies were based on forward genetics to study the function of specific genes. There were few reports to predict candidate mutant genes by reverse genetics with omics and molecular biology methods to verify that the genes are related to the biological function of concern (Zheng et al., 2022). In recent years, the development of high-throughput technology provides the possibility of non-population characteristic analysis (Shih et al., 2019). For example, Gang et al. (2019) sequenced birch leaf mutants and found that *BpGLK1* regulates chlorophyll content and chloroplast development by combining sequencing results and transcriptome data. The key candidate gene *PcMYB10.6* related to proanthocyanidin biosynthesis was identified from transcriptome data, and the underlying mechanism of constitutive activation of this gene was revealed by Gu et al. (2015). However, few researchers currently suspect that the expression level of heat shock proteins affect leaf color (Kampinga et al., 2019).

DanJ proteins belong to the heat shock proteins Hsp40 (Rosenzweig et al., 2019). The DanJ proteins consist of three domains: the highly conserved J-domain of the N-terminal, the zinc finger domain and the C-terminal domain (Kampinga et al., 2019). Currently, most researchers support the classification of DnaJ family proteins into three categories according to their conserved domains, including DnaJ-A, DnaJ-B and DnaJ-C. The DnaJ-A proteins contain a J-domain, a zinc finger domain and followed by a nonconservative C-terminal domain. Compared with DnaJ-A proteins, the DnaJ-B proteins lack a zinc finger, while DnaJ-C proteins retain only a J-domain (Walsh et al., 2004). In recent years, some scholars have discovered a new type of DnaJ proteins named DnaJ-D, which contains an incomplete HPD tripeptide of the J-domain, and its overall structure is similar to J-domain, so-called J-like protein (Zhang et al., 2018). Until now, DnaJ proteins have been identified in different species, and generally play a role in plant growth and development, stress resistance, etc (Jacob et al., 2017). With the in-depth study of heat shock proteins, their role in regulating leaf color has also been revealed (Liu et al., 2022). For instance, The DnaJ-A proteins *DJA6* and *DJA5* are essential for chloroplast iron-sulfur cluster biogenesis (Zhang et al., 2021a). Reduced expression of *OsDjA7/8* in rice leads to albino lethal at the seedling stage (Zhu et al., 2015). DnaJ-B type gene *ANU7* induces leaf chloroplast development defect, which leads to leaf albinism or partial albinism (Tamara et al., 2017). *ARC6* is a typical J-like domain gene that regulates chloroplast division, mutated *ARC6* gene could lead to fragmented chloroplast division in *Arabidopsis* (Vitha et al., 2003). To sum up, the reported DnaJ proteins related to leaf color mainly belong to three types: DnaJ-A, DnaJ-B and DnaJ-D (Zhu et al., 2015; Wang et al., 2016; Zagari et al., 2017; Chang et al., 2021). However, few studies have been reported on DnaJ-C proteins regulate leaf color.

*Catalpa bungei* is a native tree species which is famous for its precious timber and garden ornamental value, widely distributed in China. *Maiyuanjinqiu* (Identification code: 20150150) is a new variation of leaf color found in the growing seedlings of *C. bungei* by our group, which showed variegated leaves with great value in garden appreciation (Wang et al., 2019). Herein, we performed a genome-wide identification and characterization analysis of *CbuDnaJs*. The tissue-specific profiles of DnaJ family genes in different leaf color sectors were mapped according to the differential transcriptome, and the function of *CbuDnaJ49* was further verified in transgenic tobacco. This study not only provides good material for the innovative utilization of germplasm resources, but also provides an effective way for other plants to analyze the molecular mechanism of specific traits by using reverse genetics and molecular biology.

## Materials and methods

### Genome-wide identification of *DnaJ* genes in *C. bungei*

The *C. bungei* genome data was obtained from the sequencing assembly of our group (unpublished), and the protein database of *C. bungei* was established by blast-2.12.0+. HMMER3.0 was used to retrieve the *C. bungei* genome database with a threshold of 1×10^−5^. Removed the redundancy results, and the candidate members were preliminarily obtained. The candidate sequences were identified by NCBI-CDD (https://www.ncbi.nlm.nih.gov/Structure/bwrpsb/bwrpsb.cgi).

### Bioinformatics analysis of *CbuDnaJs*

ProtParam (http://www.expasy.org/tools/protparam.html) was used to analyze the theoretical isoelectric points and molecular weights of CbuDnaJ members. The subcellular location of the J proteins was predicted using Wolfpsort (https://wolfpsort.org/). The conserved domains of proteins were analyzed by Pfam (http://pfam.xfam.org/). TBtools was used to visualize the results of gene domain data from Pfam. Local blast search was used to find the paralogous genes of DnaJ family in the genomes of model plant, such as *Arabidopsis thaliana* and *Populus trichocarpa*, with at least 150 scores and powerful E-value support. With the identify similarity > 50% as restriction condition, the protein sequences of selected candidate genes were used to construct a phylogenetic tree. Multiple sequence alignment was performed using ClustalX 2.1 with default parameters. The phylogenetic tree was constructed using MEGA X (http://www.megasoftware.net.) with 1000 bootstrap tests and classified according to the homology. The MEME online program (http://meme-suite.org/tools/meme) was used to analyze protein sequences, ten conserved motifs were predicted. Gene Structure Display Server (http://gsds.gao-lab.org/) was used to determine exon-intron structures. The positions of *CbuDnaJs* on chromosomes were obtained from the *C. bungei* genome annotation files. The collinearity analysis was conducted using MCScanX software. The 2 Kb upstream nucleotide sequences of *CbuDnaJ* genes were acquired from the *C. bungei* genome data. PlantCARE online server was used for cis-elements analysis. The visualization of all the above studies was completed by TBtools and enhanced using Adobe Illustrator.

### Plant material

Leaf samples of *Maiyuanjinqiu* were collected from the long-term breeding base in Luoyang, Henan Province of China. Fresh plant leaves were immediately frozen in liquid nitrogen and then kept at -80°C for experiments. The different leaf color sectors were collected from *Maiyuanjinqiu* and *C. bungei* according to the method of Wang et al. (2019), respectively. Maiyuanjinqiu has leaf variegated character, the yellow edge was labeled as Y1, the green sectors were named Y2. The corresponding sectors in C. bungei were labeled as G1 and G2, respectively. *Nicotiana tabacum* was used as wild type (WT) in this study. Tobacco seeds were sown on Murashige and Skoog (MS) media and then transplanted in sterilized soil after germination in the artificial climate chamber. The photoperiod was 15h day/9h night, and the humidity was 40-60%.

### Expression pattern analysis of *CbuDnaJs* in different leaf color sectors

Expression patterns of *CbuDnaJs* in different leaf color sectors of *Maiyuanjinqiu* and *C. bungei* were analyzed based on transcriptome data (**Table S1**), and the heat map was visualized after log^2-^ conversion. Quantitative Real-time PCR (qPCR) was used to analysis the tissue-specific expression of *CbuDnaJ49* by Abbas et al. (2020), primers information at **Table S2**.

### RNA extraction, detection of expression levels

RNA was extracted according to the instructions for the polysaccharide polyphenol total RNA extraction kit (TIANGEN, Beijing, China) from different leaf color sectors of *Maiyuanjinqiu* and *C. bungei*, and reverse transcription carried out according to the PrimeScript™ RT reagent Kit with gDNA Eraser (Takara, Dalian, China) kit instructions. The experimental process was divided into genomic DNA elimination and reverse transcription. qPCR was performed as described by Abbas et al. (2020). The 2^-ΔΔCt^ method was used to analyze the relative mRNA expression level of *CbuDnaJ49*. Primers used for qPCR are listed in **Supplementary Table 2**. All experiments were performed with three biological replicates.

### Gene cloning and vector construction of *CbuDnaJ49* gene

The coding sequences (CDS) of *CbuDnaJ49* were amplified by PCR primers (**Table S3**). The reaction condition was as follows: 94°C for 5 min, 35 cycles of 94°C for 30 s, 52°C for 30 s, 72°C for 1 min, followed by 72°C for 7 min. The positive colonies were sequenced by Sangon Biotechnology (Shanghai), and the correct PCR products were cloned into the pEASY-Blunt Zero vector, then transferred into Trans1-T1 chemically competent cell (TransGen Biotech, Beijing, China). The full-length CDS of *CbuDnaJ49* without stop codon was amplified using primers (**Table S3**) containing the *BamH* I restriction site and synthesized into the PBI121 vector, which fused the green fluorescent protein (GFP) gene under the control of the CaMV35S promoter, this resulted in the fusion of PBI121-*CbuDnaJ49*-GFP gene. The open reading frame (ORF) region of *CbuDnaJ49* (612bp) was amplified from recombinant plasmids using primers with appended restriction sites *BamH*I/*Kpn*I (**Table S3**), and then subcloned into the CaMV35S promoter of the vector ProKII, this resulted in the fusion of ProKII-*CbuDnaJ49* gene.

### *CbuDnaJ49* transient expression vectors and subcellular localization

The vector PBI121-*CbuDnaJ49*-GFP was transformed to the *Agrobacterium tumefaciens* strain GV3101. Washed the recombinant *Agrobacterium* cultured at 28°C for approximately 24 hours and resuspended in an infiltration medium containing 50 mmol L^–1^ MES (pH 5.6), 0.5% (w/v) glucose, 2 mmol L^-1^ Na_3_PO_4_, and 100μmol L^-1^ acetosyringone. The suspensions were pressure-injected into the epidermis of tobacco leaves by using a 1mL plastic syringe placed against the leaf epidermis. The injected tobacco plants were then incubated for 12 hours in the dark, and followed by 48 hours of incubation in the light. GFP signals were detected using a confocal microscopy imaging system (Zeiss LSM880). Leaves transformed with the 35S: GFP vector alone were used as controls. All transient expression analyses were repeated three times.

### Overexpression *CbuDnaJ49* in tobacco

The constructed vector ProKII-*CbuDnaJ49* was transformed into tobacco using agrobacterium-mediated method (Asande et al., 2020). After 2-3 weeks, the adventive buds emerged, and then they were transferred to the medium with 50 mg/L *kanamycin* for inducing roots. The chlorophyll and carotenoid levels were determined according to the method of Yamatani et al. (2021). Moreover, qPCR was performed to detect the expression of genes related to pigment synthesis and chloroplast development in transgenic plants (Abbas et al., 2020).

## Results

### Identification and physiochemical properties of *CbuDnaJs*

The analysis of *C. bungei* genome using bioinformatics has identified 88 candidate DnaJ genes (**Table S4**). For convenience, the 88 DnaJ proteins were named *CbuDnaJ01* to *CbuDnaJ88* according to their position on the chromosome (**Table S5**). The average molecular weight was 45.59 kDa, and the average PI value of CbuDnaJs protein was 7.87. Among them, the isoelectric point size of the whole family of proteins was greater than 7, accounting for 66% of the total proteins, suggesting that most DnaJ proteins in the *C. bungei* were alkaline. Wolfpsort software was used to predict the subcellular location, the result showed that 21 members were located in chloroplast, 43 members were in nucleus, 9 members were in cytoplasm, 3 members were in endoplasmic reticulum, 4 members were in mitochondria, 5 members were in vacuole, and 3 members were in plasma membrane.

### Analysis of gene domains and phylogenetic tree construction based on *CbuDnaJs*

**Figure 1:**
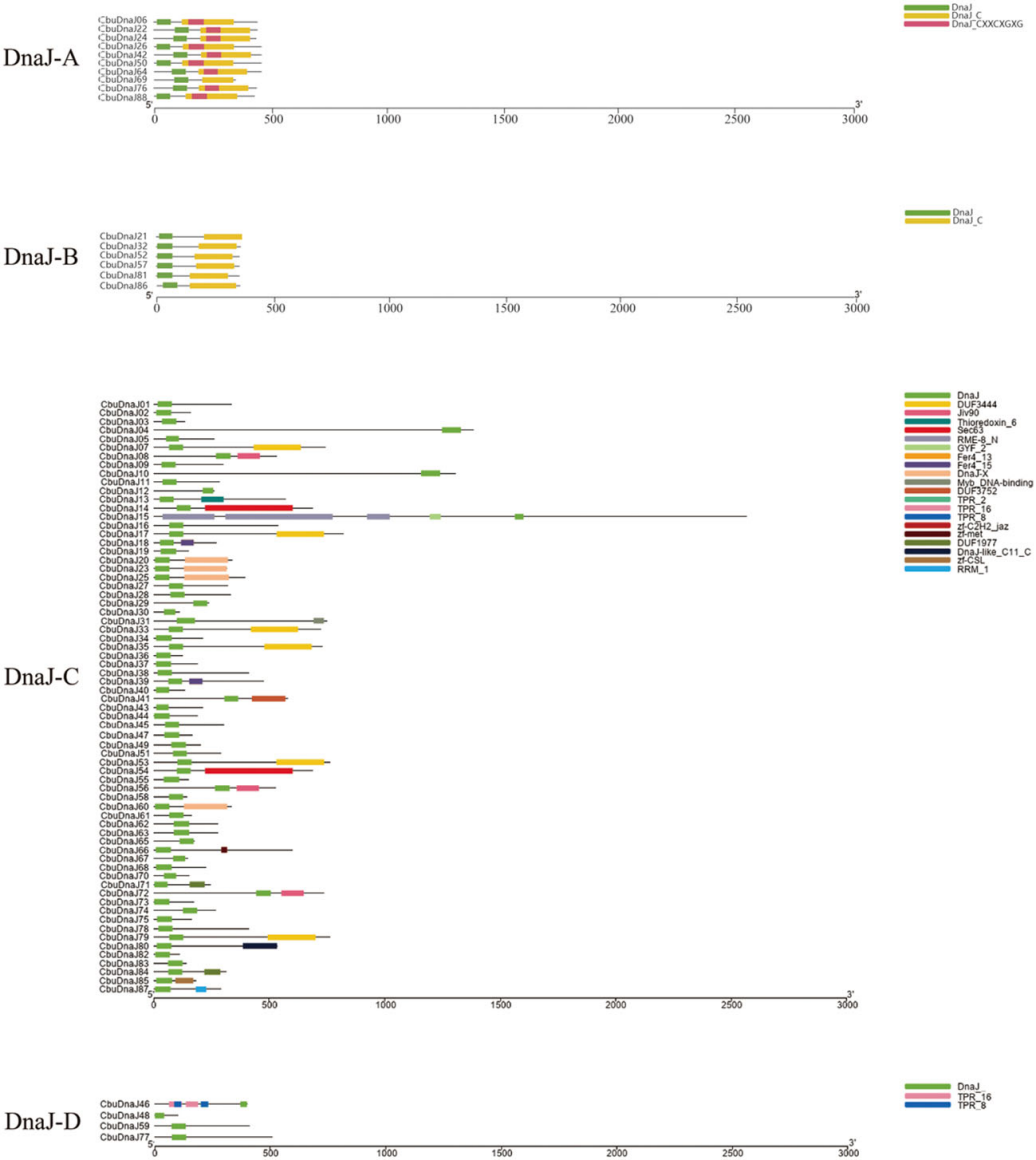
Schematic depiction of the characteristic domains of CbuDnaJs. CbuDnaJs were classified into four different types.

*CbuDnaJ* genes were classified according to the presence of the complete three domains into four types (DnaJ-A, DnaJ-B, DnaJ-C and DnaJ-D), each type has 10, 6, 68 and 4 members, respectively (**Figure 1**). Phylogenetic analysis could be utilized to predict gene function, which is important in subsequent functional studies. After setting parameters, 54 members of the DnaJ family have been identified as credible paralogous genes from *Arabidopsis thaliana* and *Populus trichocarpa*. A phylogenetic tree was constructed to comprehensively clarify the evolutionary relationship between *C. bungei* and model plants (**Figure S1**). According to phylogenetic results, each clade has members of all three species, the homologous relationship between *C. bungei* and *Arabidopsis*/*Populus* considerably matched the phylogenetic analysis.

### Analysis of gene structures, conserved motifs of CbuDnaJs

**Figure 2:**
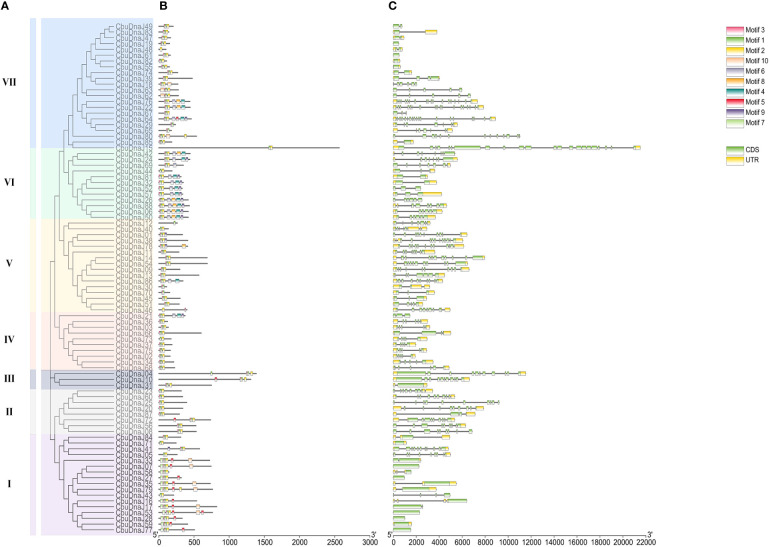
Phylogenetic relationships, gene structure and conserved motif analyses of *CbuDnaJ*s. **(A)** Phylogenetic tree of *CbuDnaJ* genes. The *CbuDnaJ*s can be divided into seven subgroups. **(B)** Motif distribution patterns. **(C)** Exon-intron distribution of the *CbuDnaJ*s. Scale markers represent gene length (bp) and protein sequence length (aa).

The exon length of *CbuDnaJ* genes in the same evolutionary branch is roughly similar but the structures differ. The result further validates the phylogenetic tree’s clustering results. To identify common motifs among the various classes of J proteins, we analyzed the motif of the CbuDnaJs. Ten conserved motifs were identified, and the distribution of these motifs in the J protein is shown in **Figure 2**. The same group of J proteins exhibited a similar pattern of motif distribution. The protein motifs in the same phylogenetic branch are identical (**Figure 2A**). To examine the structural features of *CbuDnaJ* genes, the exon-intron structures of all the *CbuDnaJs* were analyzed. The results revealed that genes in the same group shared a similar number of exons but had different exon and intron lengths (**Figure 2B**).

### Analysis of chromosomal location and duplication of *CbuDnaJs*

**Figure 3:**
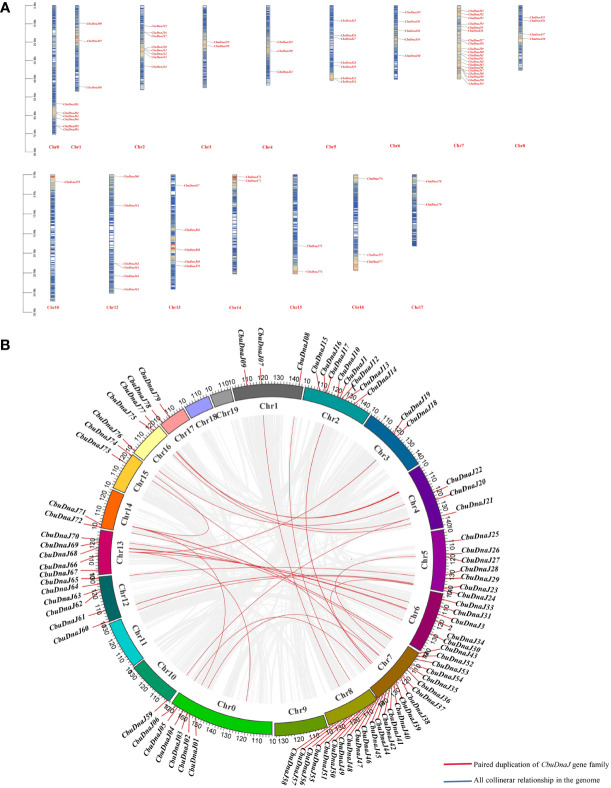
The chromosomal distribution of *CbuDnaJ* genes duplication events. **(A)** Chromosomal distribution of the *CbuDnaJ* genes. The chromosome numbers are indicated at the top end of the chromosome. The different colors in chromosomes represent gene density. The scale on the left is in million bases (Mb). **(B)** Collinearity of replicative genes in the protein family. The scale on the circle is in million bases (Mb). Gene IDs on the chromosomes indicate the positions of centromeres.

In plants, tandem and segmented duplication are the two most common causes of gene family expansion. Among the 88 *CbuDnaJ* genes, 79 *CbuDnaJ* genes were randomly distributed on all chromosomes, and 9 *CbuDnaJ* genes were not identified (**Figure 3A**). Chr7 contains the most genes, with 20 members, and the Chr10 contains the most minor genes, with only one member. The members of CbuDnaJ family genes, such as 0.2332 and 0.2339, 2.346 and 2.349, 7.3669 and 7.3670, 7.3671 and 7.3737, 12.625 and 12.627 are tandem repeat genes, indicating a large number of *CbuDnaJ* genes were duplicated to increase the number of genes and enhance their biological functions during the evolution. We evaluated tandem replication events to determine the chromosomal locus and duplication relationship in CbuDnaJ family genes. The results of collinearity analysis showed that all chromosomes had direct homologous fragment duplication events, except for chromosomes 9, 11, 18, and 19 among the 79 *CbuDnaJ* genes (**Figure 3B**). The result indicated that gene duplication event was the main driving force for the expansion of the CbuDnaJ family.

### Cis-acting element analysis of *CbuDnaJs*

We expected the cis-elements in the promoter regions 2000 bp upstream of *CbuDnaJs* using PlantCARE (**Figure S2**). The predicted results showed that the number of photoresponsive elements in the promoter region of *CbuDnaJ* genes was the largest. They followed the stress-related promoter elements, such as LTR (low temperature) and ARE (anaerobic induction). In addition, the cis-acting regulatory elements are likely involved in hormone signaling in the process of plant development because five hormone-responsive regulatory elements involved in abscisic acid, gibberellin, salicylic acid, methyl jasmonate (MeJA) and auxin response were identified respectively. In addition, we also identified cis-regulatory elements involved in palisade mesophyll cell differentiation and meristem expression, which are associated with plant developmental responses. These results suggested that *CbuDnaJs* might be involved in various biological processes.

### Tissue specificity analysis of DnaJ family in *C. bungei* and *Maiyuanjinqiu*

**Figure 4:**
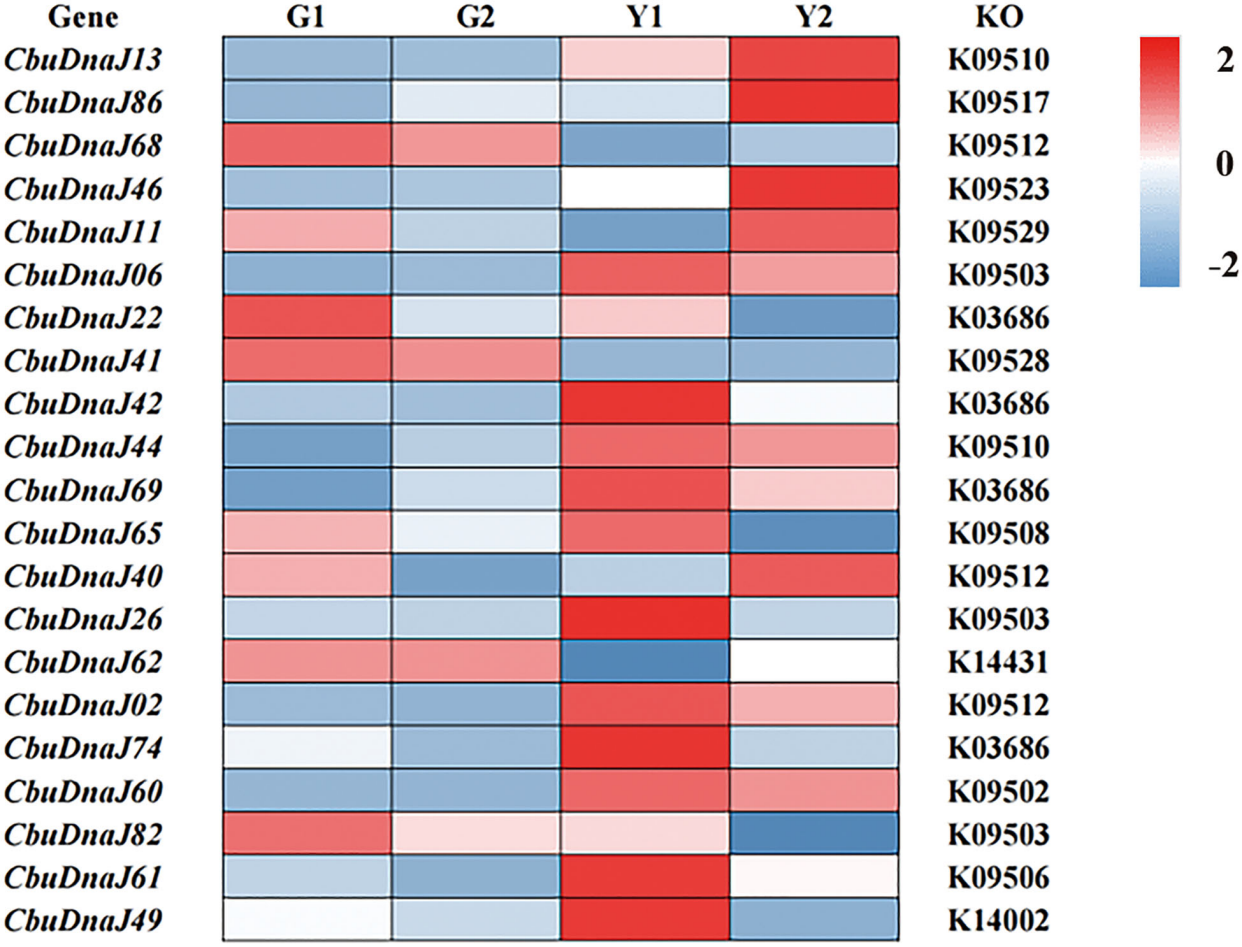
The expression profiles of *CbuDnaJ* genes in various leaf color tissues. Red and blue color scale indicates high and low expression levels, respectively.

**Figure 5:**
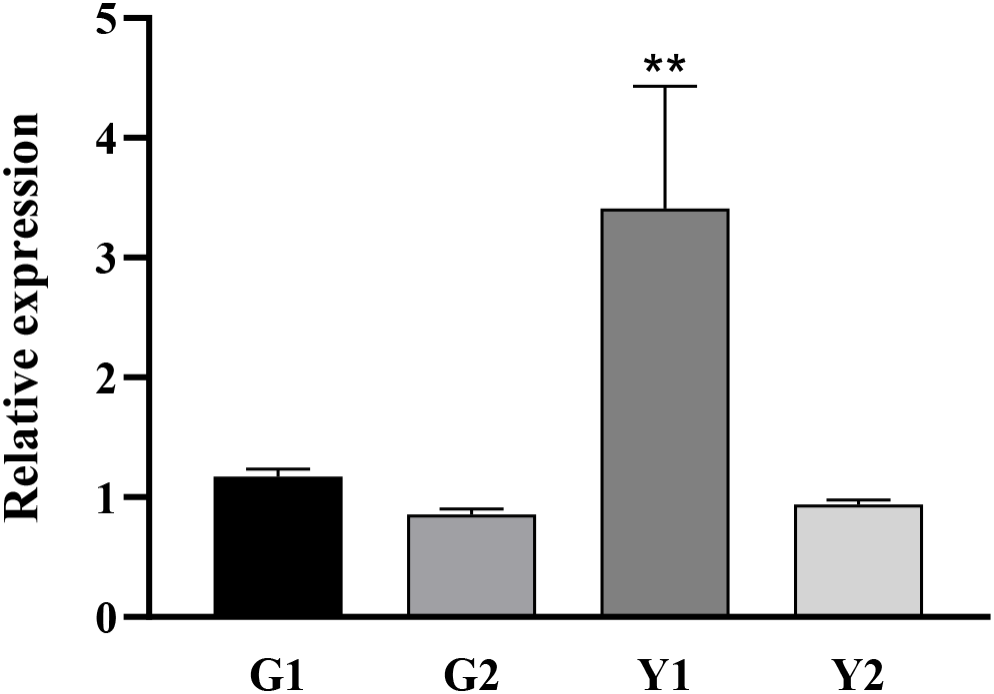
Tissue specific expression of *CbuDnaJ49* in *C. bungei* and *Maiyuanjinqiu*. Student’s t test, ** represents extremely significant difference. P < 0.01.

Twenty-two DnaJ family genes were identified and extracted from the transcriptome data of different color leaves of *C. bungei* and *Maiyuanjinqiu* (**Table S1**). The expression profiles of *CbuDnaJ* genes were illustrated in **Figure 4**. Most of the genes are expressed differentially, except for *CbuDnaJ41*, *CbuDnaJ44*, *CbuDnaJ60* and *CbuDnaJ68*. The expression levels of *CbuDnaJ02*, *CbuDnaJ26*, *CbuDnaJ42*, *CbuDnaJ49*, *CbuDnaJ62* and *CbuDnaJ74* genes were expressed in the yellow leaves differentially, while those genes were the same in the green leaves (**Figure 4**). According to the log^2-^ calculation result of RPKM, *CbuDnaJ49* is the gene with the most significant difference between the yellow and the green leaves (**Table S1**). We further compared the expression levels of *CbuDnaJ49* in different leaf colors by qPCR. The expression level of *CbuDnaJ49* in the yellow leaves was significantly higher than in the green leaves (**Figure 5**).

### Subcellular localization of CbuDnaJ49

**Figure 6:**
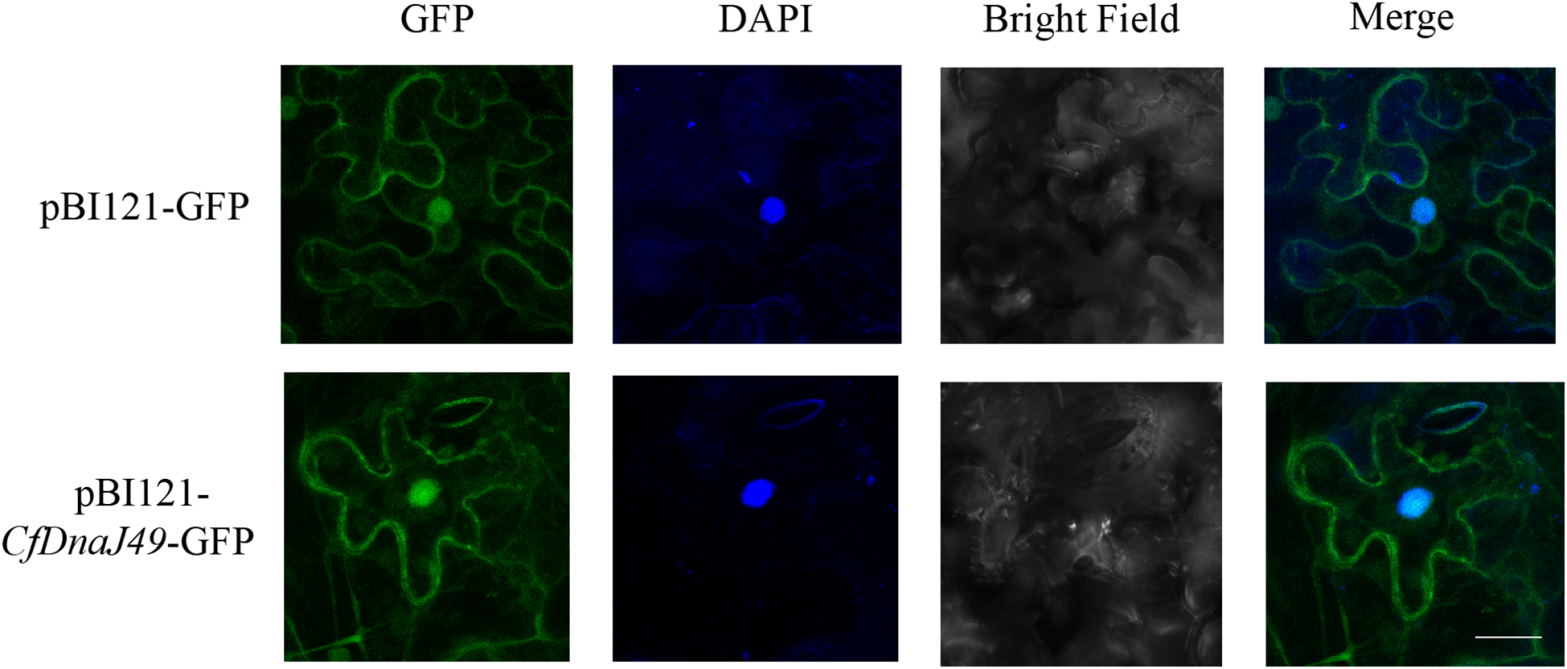
Subcellular localization of CbuDnaJ49. The length of white bar represents 10 um.

To investigate the subcellular location of CbuDnaJ49, we examined the subcellular localization of CbuDnaJ49 proteins in tobacco leaves *via* transient transformation. The constructed vectors were transferred into *Nicotiana benthamiana* leaves *via Agrobacterium* through an injection method. The CaMV35S-GFP fusion was transferred into *Nicotiana benthamiana* leaves as a control group. As shown in **Figure 6**, the control groups were primarily distributed within the cytoplasm and nucleus. The fusion proteins of CbuDnaJ49 with GFP were distributed within the cytoplasm, nucleus and chloroplast.

### Ectopic overexpression of *CbuDnaJ49* in tobacco

**Figure 7:**
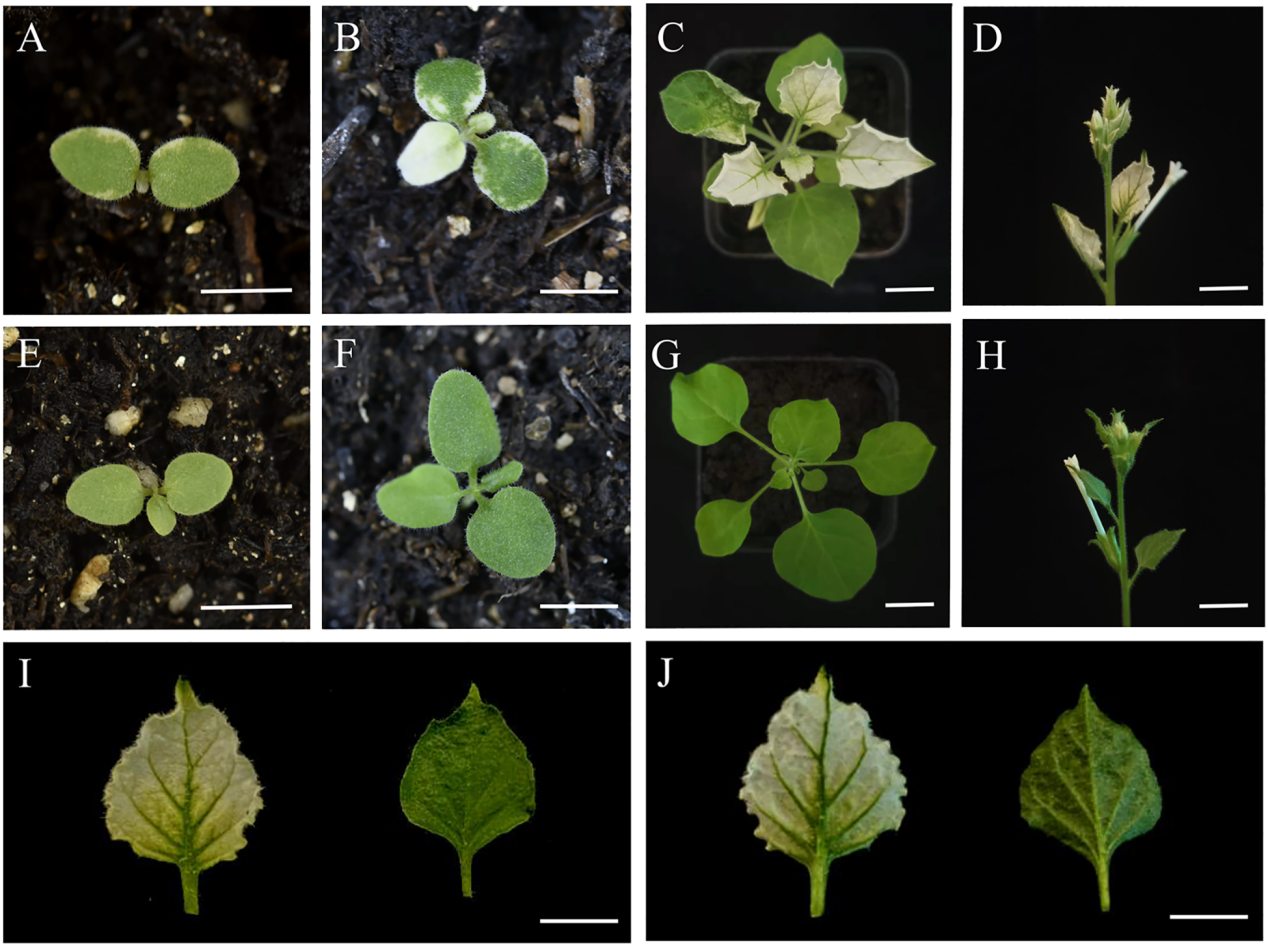
Phenotypic observations in transgenic and WT plants of *Nicotiana tabacum.* **(A-D)** transgenic tobacco. **(E-H)** Wild-type tobacco. **(I)** Upper blade surface. **(J)** Lower blade surface. The length of white bar represents 1000µm.

**Figure 8:**
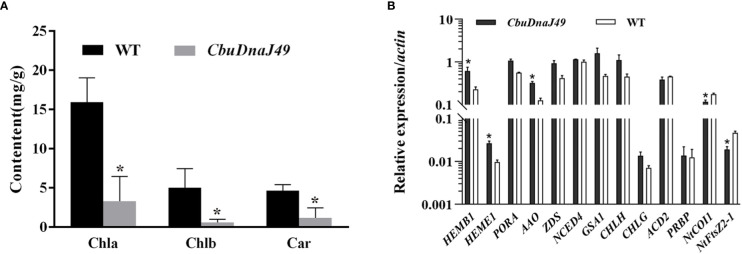
Pigment content detection and expression of the genes related to pigment metabolism and chloroplast development. **(A)** Identification and expression analysis of *CbuDnaJ49* and pigment content in transgenic and WT plants of tobacco. Student’s t test, P < 0.05. **(B)** Expression of the genes for pigment metabolism and chloroplast binding proteins. Student’s t test, P < 0.05. * represents significant difference.

Under normal conditions (25°C), leaves of transgenic plants showed leaves albino phenotype, while leaves of wild type (WT) tobacco plants showed normal green. Different from *DnaJ* mutant reported previously (Fan et al., 2017), the cotyledon and true leaves of overexpressed plants showed varying degrees of bleaching, but the veins remained green (**Figure 7**). To verify the changes of pigment in overexpressed plants, we determined the pigment content. As shown in **Figure 8A**, chlorophyll a, chlorophyll b and carotenoids in the leaves of the ProKII-*CbuDnaJ49* transgenic plants were significantly lower than those of WT. The expression levels of 12 genes involved in chlorophyll metabolism, carotenoid metabolism and chloroplast binding genes were analyzed in the transgenic plants. Compared with WT, the expression of genes for chloroplast development gene *NtFtsZ2-1* was significantly repressed in the transgenic plants. In contrast, the genes involved in chlorophyll and carotenoid metabolism were up-regulated in the transgenic plants, including 5-Aminolevulinate dehydratase (*HEMB1*), Uroporphyrinogen III decarboxylase (*HEME1*) and abscisic aldehyde oxidase (*AAO*) (**Figure 8B**). It could be inferred from the above results that the inhibition of chlorophyll binding proteins lead to the reduction of pigment content, instead of the gene expressions of chlorophyll and carotenoid metabolism pathways.

## Discussion

In this study, 88 members of CbuDnaJ were successfully identified based on the *C. bungei* genome. The identified number of CbuDnaJ family genes was lower than that found in wheat, this difference might be due to the evolutionary differences and ploidy among different species (Liu et al., 2022). The isoelectric point of CbuDnaJs protein varies from 4.64 (CbuDnaJ37) to 10.62 (CbuDnaJ61) (**Table S5**). Among them, proteins with isoelectric points greater than 7 accounted for 66% of the whole family, this result was similar to that in *Capsicum annuum* L. and *Tolypocladium guangdongense* (Wang et al., 2008; Fan et al., 2017).

The classification and nomenclature of J-domain proteins have been debated for a long time. The *DnaJ* members were initially subdivided into A, B and C types according to the bacterial *DnaJ* classification method (Cheetham and Caplan, 1998). J-domain is the defining feature of all DnaJs. It has a highly conserved and functionally critical His-Pro-Asp (HPD), which is essential co-chaperones with heat shock protein 70 (Hsp70) (Kampinga and Craig, 2010). However, with further study, some proteins with high homology to DnaJs but no conserved J-domain were found, which is characterized by the lack of crucial classification is outdated. Liberek et al. (1991) proposed that the classification of DnaJs should be based on evolutionary correlation. They suggested that even DnaJ-A within the most conserved prototypical, their function has evolved following gene duplication. In our evolutionary analysis of *CbuDnaJs*, we also found this situation (**Figure S1**). However, because there is no precise new classification method at present, we still classify it according to the structural feature method of Cheetham and Caplan (1998). But considering that the related proteins lacking the functional J-domain have essential value in the evolutionary history and functional diversification, we have not eliminated this type of gene (*CbuDnaJ46*, *CbuDnaJ48*, *CbuDnaJ59*, *CbuDnaJ77*), and classified them into DnaJ-D according to Walsh et al. (2004) (**Figure 1**). This result was similar to the evolutionary distribution of *Sorghum bicolor* (Nagaraju et al., 2020), suggesting that gene drift events occur between members of adjacent evolutionary branches within the species. The results of chromosomal location and duplication analysis showed that *CbuDnaJs* were unevenly distributed on the 20 assembled chromosomes of *C. bungei*. There were a large number of fragment repeats and several tandem repeat events in CbuDnaJ family genes (**Figure 3**), which suggested that some members of *DnaJ* gene family were evolved through gene replication events in *C. bungei* (Abdullah et al., 2021; Musavizadeh et al., 2021).

The intron-exon structure could help to understand the conservatism and evolutionary characteristics of gene families more systematically and comprehensively (Li et al., 2020). It is an important clue for the evolution and functional analysis (Pulido and Leister, 2018). The visual analysis of intron-exon structure in this study showed that the number and distribution of introns and exons were highly conservative, and most *CbuDnaJs* contain 1-3 introns. The results of conservative motif analysis also support this point (**Figure 2**). This structurally similar evolutionary branch suggested that these genes might have similar physiological functions (Wang et al., 2022). In our analysis of the vascular plant *C. bungei*, we found that the introns of almost all the DnaJ members in *C. bungei* were at both ends of the whole gene sequence, which was most likely due to the large loss of introns that occurred during the evolution of the species. Introns increase the length of genes and increase the frequency of recombination between genes, which was conducive to the evolution of species (Poverennaya and Roytberg, 2020). Walter Gilbert suggested that protein-coding genes were produced by exon integration, a process that exons together through recombination of intron sequences. Introns are thus seen as remnants of a process that promotes protein evolution (Gilbert et al., 1997). However, some other researchers support the point that the presence of introns in the genome is an energy burden for many cells (Lynch, 2002). This lost structure might help to quickly activate gene expression in response to environmental stress in *C. bungei.* The result is supported by Lee et al. (2023). In addition, promoter is an important part of genes, which can control the start time and expression degree of gene transcription, the analysis of promoter is the premise and basis of expression regulation research (Valassakis et al., 2018). We analyzed the cis-acting elements of the promoter of the CbuDnaJ family genes (**Figure S2**). The results showed that *CbuDnaJs* contained a large number of cis-regulatory elements related to plant growth hormone (gibberellin GARE-motif, abscisic acid ABRE and auxin TGA-element), defense against stress (jasmonic acid CGTCA-motif, salicylic acid TCA-element, low-temperature LTR) and light response (GT1-motif, G-box, Box 4). Several active elements, such as meristem expresses CAT-box, O2-site and HD-Zip 1 were found. These results suggested that *CbuDnaJs* might play a role in regulating various biological activities.

Subcellular localization results showed that the CbuDnaJ49 was expressed in chloroplast, nucleus and plasma membrane. This result was different from that of the homologous gene *AtDnaJ20* in *A. thaliana* (Chen et al., 2010), suggesting *CbuDnaJ49* might have new functions (**Figure 6**). Ectopic expression in model plants is a common experimental method to identify gene function due to the lack of regenerative and transgenic systems in woody plants (Abbas et al., 2020). To explore the biological function of *CbuDnaJ49* gene, the overexpressed *CbuDnaJ49* gene was transformed into tobacco, and the pigment contents in transgenic tobacco leaves were measured to identify the positive transgenic plant. The leaves of overexpressed plants showed different degrees of bleaching phenotype, and a few plants started from the cotyledon, and the leaf edge showed chlorosis (**Figure 7**). In our study, the chloroplast development gene FtsZ was significantly down-regulated in transgenic plants, the down-regulation of FtsZ has been reported in tobacco to disrupt normal chloroplast development severely (**Figure 8B**) (Vitha et al., 2001). In contrast, the expression levels of genes related to pigment metabolism did not significantly reduce in albino leaves, there might exist a compensatory mechanism in the albino leaves, such as post-transcriptional regulation, however, the detailed mechanism remains to be further studied (Song et al., 2018). Although previous studies have shown that several *DnaJ* genes played a pivotal role in chloroplast assembly and division, however, these reported genes usually belong to the type of DnaJ-A, DnaJ-B or DnaJ-D (Chiu et al., 2010; Dorn et al., 2010; Chen et al., 2011). The identified gene *CbuDnaJ49* is a member of DnaJ-C that contains only J-domain, until now, no such type of *DnaJ* genes associated with leaf color has been reported. Although *CbuDnaJ49* was presumed to be a key gene in regulating leaf color, it is not yet clear that the mutation of this gene was responsible for the leaf color character of *Maiyuanjinqiu*. It is not impossible to obtain effective information by mapping cloning, the combination analysis of multiple omics might be helpful to further analyze the molecular mechanism of leaf color formation in *Maiyuanjinqiu.*

## Conclusions

Here, we performed a genome-wide identification and characterization analysis of DnaJ family genes in *C. bungei* for the first time. We identified the largest differentially expressed gene between the green and yellow sectors of *Maiyuanjinqiu* and *C. bungei*. Furthermore, we verified that *CbuDnaJ49* was indeed related to leaf color by ectopic overexpression in tobacco. The transgenic plants exhibited albino leaves, and the contents of chlorophyll and carotenoid were significantly reduced compared with those of wild type. The identified gene *CbuDnaJ49* is a member of DnaJ-C that contains only J-domain. Until now, no such type of *DnaJ* genes associated with leaf color has been reported. This study not only provides good material for the innovative utilization of germplasm resources, but also provides an effective way for other plants to analyze the molecular mechanism of specific traits by using reverse genetics and molecular biology.

## Data availability statement

The original contributions presented in the study are included in the article/**Supplementary Material**. Further inquiries can be directed to the corresponding author.

## Author contributions

NW and YY conceived and designed the experiments. YY performed the experiments and data analysis and wrote the original draft. NW directed the experiment and revised the paper. LZ, JM, YZ, PF, CY, and JH provided help in the experiment. NL and WM contributed to the data analysis. LZ and YZ sampled the materials. All authors contributed to the article and approved the submitted version.
