# Supplementary material for: Genome-wide identification of DnaJ gene family in Catalpa bungei and functional analysis of CbuDnaJ49 in leaf color formation
Source: Front Plant Sci. 2023 Feb 23;14:1116063. doi: 10.3389/fpls.2023.1116063 (PMC10038198; doi:10.3389/fpls.2023.1116063)
Supplement: Supplementary file 1 [file DataSheet_1.docx]

Supplementary Material

Genome-wide identification of DnaJ gene family in *Catalpa bungei* and functional analysis of *CbuDnaJ49* in leaf color formation

**Yingying Yang^12^, Linjiao Zhao^3^, Junhui Wang^1^, Nan Lu^1^, Wenjun Ma^1^, Jiang Ma^2^, Yu Zhang^1^, Pengyue Fu^4^, Chengcheng Yao^1^ , Jiwen Hu^1^, Nan Wang^1*^**

*** Correspondence:**

Corresponding Author: [nanwang2019@caf.ac.cn](mailto:nanwang2019@caf.ac.cn)

# Supplementary Figures and Tables

## Supplementary **Figure 1.**


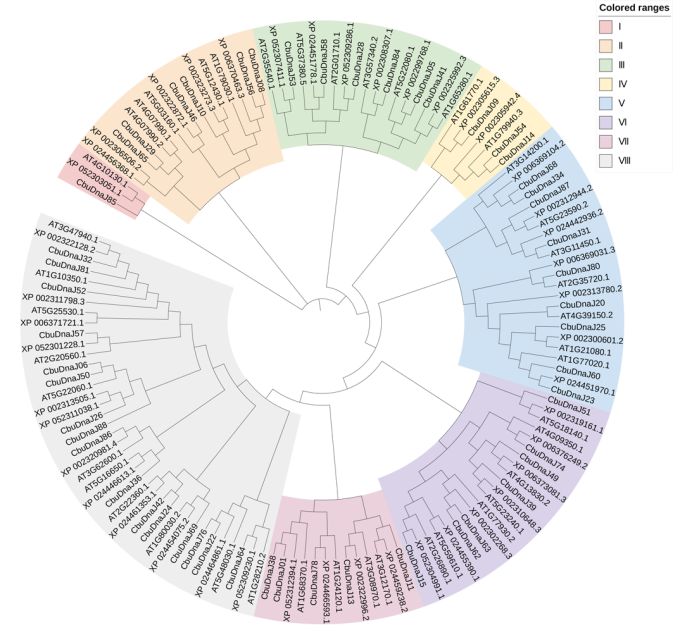


Phylogenetic tree of J-proteins of *Catalpa bungei, Populus trichocarpa* and *Arabidopsis*.

## Supplementary **Figure 2.**


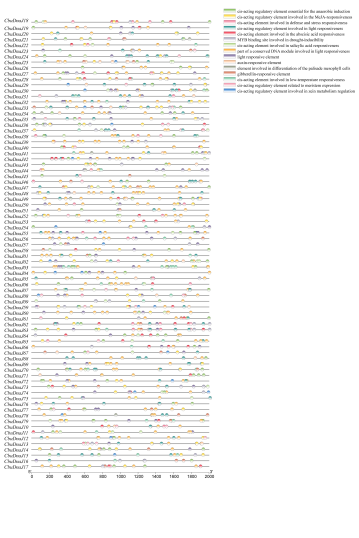


Promoter analysis of *CbuDnaJ* genes.

## Supplementary Tables

### Supplementary Table 1.

*DnaJs* transcriptome data in different leaf color sectors of *Maiyuanjinqiu.* and *Catalpa bungei.*

| **Gene_ID** | **FPKM.G1_7** | **FPKM.G1_8** | **FPKM.G1_10** | **FPKM.G2_7** | **FPKM.G2_8** | **FPKM.G2_10** | **FPKM.W1_6** | **FPKM.W1_7** | **FPKM.W1_8** | **FPKM.W2_6** | **FPKM.W2_7** | **FPKM.W2_8** |
| --- | --- | --- | --- | --- | --- | --- | --- | --- | --- | --- | --- | --- |
| *CbuDnaJ13* | 2.4234 | 3.0366 | 2.7184 | 1.9448 | 3.1487 | 3.2304 | 2.4698 | 5.5017 | 2.9538 | 5.0124 | 5.9699 | 2.3407 |
| *CbuDnaJ86* | 20.6271 | 18.8122 | 22.8328 | 23.3899 | 19.8435 | 24.6722 | 16.7922 | 31.3251 | 18.7817 | 32.6358 | 32.8860 | 17.6342 |
| *CbuDnaJ68* | 3.8370 | 2.1794 | 5.5200 | 1.8130 | 2.3042 | 5.6807 | 0.6959 | 0.1415 | 0.0000 | 0.2801 | 1.0187 | 1.6316 |
| *CbuDnaJ46* | 13.6672 | 9.6694 | 16.2220 | 13.8965 | 12.9438 | 13.3005 | 15.2358 | 16.6497 | 12.9283 | 21.0499 | 19.7946 | 14.0761 |
| *CbuDnaJ11* | 4.3803 | 4.5824 | 8.1491 | 3.0774 | 4.1670 | 8.2971 | 3.8936 | 6.8548 | 3.9009 | 6.4136 | 7.5704 | 3.9749 |
| *CbuDnaJ06* | 102.5609 | 94.6435 | 106.2115 | 95.9556 | 108.0373 | 111.9859 | 142.2929 | 205.9194 | 165.3790 | 148.1778 | 151.0060 | 167.4622 |
| *CbuDnaJ22* | 10.6771 | 10.8658 | 11.4853 | 9.0781 | 10.9980 | 9.8471 | 8.4928 | 12.8425 | 9.9796 | 8.9556 | 8.2430 | 10.9593 |
| *CbuDnaJ41* | 28.1016 | 24.8315 | 24.9862 | 25.3065 | 26.2127 | 22.1771 | 17.9504 | 14.6361 | 15.5787 | 14.5521 | 15.2293 | 17.9548 |
| *CbuDnaJ42* | 74.5100 | 78.3618 | 63.0669 | 81.0841 | 74.4385 | 57.3172 | 86.5493 | 85.8479 | 98.4389 | 73.4884 | 81.9485 | 75.7492 |
| *CbuDnaJ44* | 5.8747 | 4.4029 | 4.4348 | 6.5577 | 5.2951 | 6.3180 | 9.6932 | 7.6481 | 10.8081 | 8.8636 | 9.2838 | 7.9315 |
| *CbuDnaJ69* | 29.0138 | 34.1579 | 34.3412 | 35.4841 | 37.6059 | 37.5362 | 49.3247 | 43.3107 | 46.8005 | 43.8974 | 39.6167 | 40.3615 |
| *CbuDnaJ65* | 17.3841 | 18.1501 | 14.0917 | 14.7602 | 16.8186 | 13.0221 | 16.6050 | 19.3449 | 17.6355 | 12.3172 | 11.3174 | 11.9857 |
| *CbuDnaJ40* | 28.3771 | 27.7203 | 27.2986 | 22.3835 | 27.2967 | 23.1183 | 26.4553 | 26.7617 | 23.1999 | 31.5300 | 34.5776 | 21.1840 |
| *CbuDnaJ26* | 728.6949 | 703.5724 | 763.9450 | 633.5255 | 762.4663 | 787.6927 | 1049.9066 | 841.0519 | 990.2269 | 675.8813 | 635.1597 | 879.7932 |
| *CbuDnaJ62* | 9.3017 | 11.3680 | 7.4356 | 10.3119 | 11.0958 | 6.7412 | 7.4840 | 4.2342 | 7.4953 | 8.0893 | 7.7948 | 9.2746 |
| *CbuDnaJ02* | 25.2125 | 19.6419 | 21.0233 | 21.0134 | 20.0173 | 17.3677 | 61.0715 | 87.3330 | 95.5082 | 66.5573 | 52.8185 | 66.0542 |
| *CbuDnaJ74* | 111.3325 | 112.0243 | 66.9638 | 88.5778 | 95.8295 | 63.4811 | 139.7553 | 105.3660 | 142.9498 | 75.3126 | 74.3411 | 115.4129 |
| *CbuDnaJ60* | 5.6699 | 4.2421 | 4.3482 | 4.5032 | 6.0386 | 3.5846 | 14.0468 | 7.2068 | 11.5665 | 13.2980 | 6.9775 | 9.5639 |
| *CbuDnaJ82* | 26.1693 | 54.3216 | 33.0012 | 25.3583 | 32.6078 | 22.2106 | 47.2459 | 13.4313 | 20.4112 | 1.1005 | 1.7792 | 2.1010 |
| *CbuDnaJ61* | 13.6456 | 16.7028 | 8.6042 | 13.5470 | 10.6007 | 8.2321 | 25.8248 | 21.8232 | 22.2636 | 16.2489 | 18.3899 | 13.4917 |
| *CbuDnaJ49* | 79.7141 | 65.9090 | 94.3376 | 23.6791 | 34.3121 | 51.9489 | 100.7506 | 98.7802 | 125.3524 | 2.2122 | 1.5844 | 2.8545 |

### Supplementary Table 2.

Primers used for qRT-PCR

| **Primer name** | **Upstream primer（5’-3’）** | **Downstream primer（5’-3’）** |
| --- | --- | --- |
| *HEMB1* | ATGAGCTTTTGCAAAATTTCTTCATC | TCATCTCTGAGCTCTCATTCGAGC |
| *HEME1* | ACGGGGGACTCTAGAGGATCCATGAGCTTTT GCAAAATTTCTTCA | GGATCTGACCACCCGGGGATCCTCTCTGAGCTCTCATTCGAGCA |
| *PORA* | ACGGGGGACTCTAGAGGATCCATGAGCTTTTGCAAAATTTCTTCA | GGGAAATTCGAGCTGGTACCTCTCTGAGCTCTCATTCGAGCA |
| *AAO* | GACACAGACTGCCCTTGCGA | CTGACTGCCTTGCCCCCAAA |
| *ZDS* | TGCAGGACTTGGAGCGTTCG | TGTACGGGTCACCTGGCGTA |
| *NCED4* | CCACGTTACGCCAAGGACGA | CTCCAGCGTATGCTCCACCG |
| *GSA1* | TAGGACTCCCCGACTCCCCT | GCTCCGCCATATGCCAACCT |
| *GHLH* | GGGGCACCGAGCTGAACTTT | AGGTGGCTGCTGGTTTGCT |
| *GHLG* | GCTGGAACGTGTGGTGGTGA | AGTGTTTGCATCCCGCCAA |
| *ACD2* | GGCCACTCCTCTTCCCAGGT | TGTTGGACTGCTGCGGATGA |
| *PRBP* | TAGGATGGGTGGTGGCCCAA | TTTGGCGTTCCCGAGCCAAT |
| *NtCOI1* | CGGCTCAGGATTGAGCGAGG | CGGTCAAGCAAAACCAGCCG |
| *NtFtsZ2-1* | GGCCCCTCCAGGTAGTCCTC | GCGAGAGCCCCCTTTCTTCC |
| *actin* | TGTGTTGGACTCTGGTGATG | CGCTCGGTAAGGATCTTCATC |
| *qPCR-CbuDnaJ49* | TCTTCCCAAATTCGAACCAG | CCCAGCAGTTCGTACAGTGA |

### Supplementary Table 3.

Primers used for PCR

| **Primer name** | **Upstream primer（5’-3’）** | **Downstream primer（5’-3’）** |
| --- | --- | --- |
| *CbuDnaJ49* | ATGAGCTTTTGCAAAATTTCTTCATC | TCATCTCTGAGCTCTCATTCGAGC |
| pBI121-*CbuDnaJ49*-GFP | ACGGGGGACTCTAGAGGATCCATGAGCTTTT GCAAAATTTCTTCA | GGATCTGACCACCCGGGGATCCTCTCTGAGCTCTCATTCGAGCA |
| ProKII-*CbuDnaJ49* | ACGGGGGACTCTAGAGGATCCATGAGCTTTTGCAAAATTTCTTCA | GGGAAATTCGAGCTGGTACCTCTCTGAGCTCTCATTCGAGCA |

### Supplementary Table 4.

DnaJ amino acid sequence of *Catalpa bungei.*

| **Sequence ID** | **Amino acid sequence** |
| --- | --- |
| evm.model.group3.897 | MEVSMSMSMYKNSKPIISCKLVALQSGKTNFYQLLSLSSQNVGFDEIKKAYKTMILRYHPDVCNRSVLTKEESTKRFIELRKAYETLSDPNARRVYDYELMGLEDPFGAHFGAFCGEKRRESLSREVWELQLNGLRKKSVERMKKRRSYNCS* |
| evm.model.group3.1209 | MENRKRLKRQKSVLVRCNHEKLGGSKNHYELLGVSIDASQQEIKGAYRKLQKKYHPDIAGEEGHERTLMLNKAYKVLIRDDLRKEYDASIGRVSDGVGFGKGGYGSIWKGPLRPQALFVDENACIGCRECVHQASNTFVMDEAFGTARVKVQYGDDDGKIEISVASCPVNCIHLINTEELRVLEYLIQPQPKPGYGIYGQGWERPAHVFMAAKSFSKQLKQQEENQQKYAQSRDEEEETPAQAQARESAYRELKLGSFARIWSWIKKNTGQ* |
| evm.model.group15.872 | MVPFLNPKFRPPNTFSLSSMAAPTATPPSSFSLSLRTLTSKPSIHLIKLRKRRISHHGCLGVVAVQGSSGRQRPPPGVDTRIHWENEDEGWIGGSNSRSTQEQEKEEEELLGEKFSDLLESSSDSYYQFLGVSASADLEEIKAAYRRLSKEYHPDTTTLPIRTASDKFMKLREIYDVLSDDEKRRFYDWTLAQEAASREAEKMRMKLEDPYMQEVENWESIPDMVDRLGGRNMELSDQAKQALTFDFLIIIFSICCIIYTVFFKEANY* |
| evm.model.group15.557 | MEYYSVLGVNTDASDEEIRRAYRKLAMQWHPDKWTRSPSHLGEAKQKFQQIQEAYSVLSDGRKRMLYDAGIYDRDDEEDEIDGFADFLQEMASLVHEARKEEKNYSFEELQRMFWEMTQDFEMNNSMQQPTYGSHWFCGPFSATSDSWEVSMTQGNPHTNKLSFETYGAHHCCR* |
| evm.model.group2.1016 | MSPPVTNQYPTVEISKTNCSLTQNLESSGSFNFAAGLTDAAGFNYSKSGPQKPNHSCRSRPRLVKVRKKQTATSASQDGKSVKTDLGLNGFGDVSGDTTKFDGQLGNVVGGNNRNNGSANNADLNGNVEQRRNGFGFGVSLNESLFGLGLGNGKSFFGSSMNTSTTNSHSKEGDFLFASNKGCSNLDVQKESGSFVFGAHEAGSTANKKSDNIEFVFDSDMKKDQQDSNVSGFHFSVDEFEKVNSAKFVFGASKNASTVINSDQQKEDRSKSMDKSEFDKDVGNTIPDIRGKVKLDASGDSGKVCHPSFEFPFNLSDNSSKNDVNFVFGSNNIDSKPEIDPANNPIGKNRDMSNFTNSSEENAEIDIQFQNACLNGVFVFGGLKGKGCLNSGGSTKLVNEMNQSNRGKTEDCNGFGQHNHDTGSDINSKSSTSSGCSFENPAFGISDVMKRLNIGDCEGDANKTENFNNNFSVNTNAFVFGKDQKNSDFMKENHGGSIGQKTPEVSHLSHNNSETNQSSSLMFPSVGIGIHLNGGFCETPSMNKDEKDSISFTGKLAGSDADYSTNMEFAFSNPFPGSDRKLDNANSKSLGGTRLKKKNGKLRQRTMVQQLFGQDRVSKDGNTQQNQSPGCGSPMDFSPYHDTCANNAPDADTGTEVKVEFASKERDNLENSGNSHDDGSNSNFSPSLPAQDGLSAVRHQYKKKYKLKIGSNHTVQSNNSDKENVKQEPLGAASHEVCEHWRIRGNEAYHAGKLSKAEEFYSIGINSVTHVSALGFFIKPILLCYSNRAATRMSLGRMREAIGDCTKAAELDPDFLKATLRAGNCYLVLGEVEDAIQCYTKCLSSGIGVCLDRRVTIEAADGLQKAKRVAEYMQQSAKLMQEGTEDAASNALGNIADALSISRYSDKLLEMKGKALYILRMYDEVIQLCDQTLDIAIKNFGADHFDDPSCKRSHVKFWRWHLQAKSHYHLGKLDLALDLIEKHEEMPISSKSGDTTGEALIALAATIRELLSLKRSGNEAFNSGRYTEAIENYTSAISKSFESRPFMAICFCNRAAAYQAIGQIVDAIADCNLAIALNENYQKAISRRATLHEMIRDYKQAVSDLQRLISLLESRSLSKSQQQDSSRSSAGSVRDLRKARRHLSSIEEKAKKETPLDLYLILGIKASDVESEIKKAYRKAALRHHPDKAGQILGRSDVGDDGTLWKEIGEKIHNDADKLFKIIGEAYAVLSDPSKRSKYDDEEEMRNIYRDSNRNSNAGHPSTSYNSQYERGSWYGRQAGYSTSFERNNSRRHWYDSRSYGTSNQW* |
| evm.model.group2.346 | MECNKDEAIRAKEIAEKKMENNDFEGARKIALKAQNLYPELENITQLLSICDVHCSAQKMLLGSDKDWYGILQVEKLADELMVKKQYRRLALILHPDKNRFPGAEGAFKLICEANAVLSDPTKKSLYDNKIKVSVRTAPVNPPHHQINRTSQLNKQYSAPNNVSNGFSSINQRPATQSTFSVRQDIFWTSCPFCSVRYQYFRQFVNKSLRCQTCSKVFIGYEITAQGVSLGSKSGQPVPQYVPAKCDSSRPAGFQEKVGANQSNCKMGVQKNKESSQSHAGSQGISNGKAVRLEPGVNTGSGSEGAAKVSGDLKAKETGIRNANSFHGGKEGGTSNGDTINRETRDLKNKRRKRGRKVVESSESCDTSGDSDLEDVTMDGNFDDLASDLKPGSGHVHFPRRSSRKRQSVSYKENDEDDIASSVKGVQATNEDNAKEQKGNVGTEDLKHGNHNGFPTDADCSKSETEERGTVHSGETLRSKDADCDKGVKVRGETGHKSGIGADTVEIESDSDRVSISSDDNPNVDLCHCPDPEFSDFD |
| evm.model.group2.1856 | MAASEENSALFPIFVLTIIALPLVPYTIVNVYLTFRKKIKNLNCRCSVCSRSGKYRKSIFKRISNFSTYSNLTVVLLWVVVVLLVFYIKHIDAEIKIFEPFSILGLESGASDSEIKKAYRRLSILYHPDKNPDPEAHKYFVDYISKAYQALTDPVSRENFEKYGHPDGRQGLQMGIALPQFLLNIDGASGGILLLGIVGVCIILPLTIAVIYLSRSSKYTGNYVMHHTMYAYYHFMKPSLAPSKVMEVFIRAAEYMEIPVRRTDEESLQKLFMLVRSELNLDLRNIRQEQAKFWKQHPALVKSELLMQAHLTRETTALSPYLQLDFKKMLALAPRLLEELMKMAIMPRTQQGHGWLRPAIGVIELSQSIIQAVPLSARKATGASSEGYGPFLQLPHFSESVVKKIARKKVRSFQDLREMKPQERAELLTQVAGFSADESQDVEVVLEMMPSISIDITCETEGEEGIQEGDIITMQAWVTLHRWNGVIRALPHAPYFPYEKEENFWLLLADSSSNDLWMSQKVNFMDEATAIIAASKAIQELKEGSGASAKEINAAVKEAIDRVKAGSRLVMGKFQAPSEGNYNLTSYCLCDSWISCDAKTNIRLKVLKRSRAGTRNIAAADEVPLLDDGAEEEDEEGEDEYDDDYESEYSEDDEDVKGKDAKGAVANGSVYAGNGSSSDGSESDDN* |
| evm.model.group2.349 | MECNKDEAIRAKEIAEKKMENNDFEGARKVVLKAQSLYPELENITQLLSICDVHCSAQKRLLGSEKDWYGILQVEKLADELMVKKQYRRLALILHPDKNRFPGAEGAFKLICEANAVLSDPAKKSLYDSKIRVSVRSAPVYPPHHQINKNSQLNKQYGAQNNVSNGFSSRNQHPATQSTFSTSKNVFWTSCPFCSVRYQYLRQFVNKSLRCQTCSKLFIGYEISTQGVSEGSKSGQPAAQYAPPKPDSSKPAGFQEKVVQNQGNSKMSVQNKKGSSPSHARSQGIASRETVRPEPGVHTGSGSEYVQEASAAKVSGDSKPKQTDTCNANSFQEGKKGGTSNGDAINRETRDLKNKNKKGRKLEESSESSDTSSDSDLENVTMKENSGDPADLKSESGHVHIPRRSSRKRQRVSYDEKEEDDLASPVNGEQTAEENNGKEQKDAVESEDTKHSNNNNPPTDVDYSKPKTKERETVPSEESLKSKNADYDSKVKLRGEIGSKSGISGIGADTIEIESDSEQVSVSSDNHEADLCHCPDPEFSDFDKERDESRFEVNQFWACYDTLDGMPRFYAKVKKVHLSPFELSITWLEAVPMDEAYKKWVDEELPVGCGSFKLGKTEKTSARLSFSHQVHCEKGKKRGSLIIYPREGEVWALLKNWDLSWSSNPENHEEFKYEMVEVLSDFVAGAGIKVGYLDKVTGFVSLFKRASRSVTDSFFIRPDELYKFSHRVPSFKMTGSEREGVPVGSFELDPAALPMNSDDVYCPGKVKLEFENVDPGVHCSPPKSAKKKGKSAVSEDTNTPKKFVDLEGIKDSNLSSKAVTDL* |
| evm.model.group2.203 | MDFVSRHAASPSDHAPPTPSASSSTPAAPPEEPEYVARYMVIKHSWRGRYRRILCISNYAIITLDPNTLSVTNSYDVGSDFEGAAPIIGRDENSNEFNISVRTDGRGKFKAIKFSSKYRSSILTELHRIRWSRLATVAEFPVLHLRRRTSEWVPLKMKVTYVGVEVIDLKSGDLRWCLDFRDMDSPAIVLISEAYGRKNVDQGGFVLCSLYGRKSKAFQAASGTSNAAIISNLTKTANSMVGVSLVVDKSQSLTISEYMKQRAKEAVGAEETPLGGWSVARLRTAAHGMLHSSGLSLALGPKGGLGDSGDAVSRQLILTKVSLVERRPENYEAVIVRPLSSVSALVRFAEEPQMFAVEFNDGCPIHVYASTSRDSLLAAVLDVLQTEGQCPVPVLPRLTMPGHRIDPPCGRVHLQYPQQLQRPVTDMESATMQLKHLAAAAKDAVAEGGSIPGSRAKLWRRIREFNACIAYSGVPTSIEVPEVTLMALITLLPATPILPPESLPLPPPSPKAAATVMGFLACLRRLLASKSAASHVMSFPAAVGRIMGLLRNGSEGVAAETVRLIALLVGGGPGDTNMLSDTKGEQHATIMHTKSVLFAEQSNLTVLVNRLKPISVSPLLSMAVVEVLEAMICEPHSETTQYTVFVELLRLVAGLRRRLFALFGHPAESVRETVAVVMRSIAEEDAVAAESMRDAALRDGALLRHLLHAFYLPAGERREVSRQLVALWADSYQPALDLLSRVLPPGLVAYLHTRPNGISADEVSNKEVSLMSRRQRRLLQQRRNRPVKGIPSQGQSMPSVNDAEGTDQAPQKSGYAFRDLDGNRSTAGDPNIGKVPSVISSVVHGVENFSNDLPVVAVPPNNQSSVISSPDKYAVHASELIETNATNANDTDIGASGLQNSGIPAPAQVVIENAPVGCGRLLLNWPDFWRAFGLDHNRADLIWNERTRQELMEALQAEVHKLDIEKERTEDIVPGGVSKETMGGQETVPQISWNYTEFSVRYPSLAKEVCVGQYYLRLLLESGTSGRAQDFPLRNPVAFFRALYHRFLCDADTGLTVDGAVPDEMGASDDWCDMGRLDGFGGGGGSSVRELCARAMAIVYEQHSNTIGPFEGTAHVTVLLDRTNDRALSNRLLLLLKVLMKVLSNVEACVLVGGCVLAVDLLTVVHEASERTAIPLQSNLIAATAFMEPLKEWMFINKDNAQVGPVEKDAIRRFWSIKEIDWTTRCWASGMPDWKRLRDIRELRWAMAVRVPVLTQIQVGEAALSILHSMVAAHSDIDDAGEIVTPTPRVKRILSSPRCLPHIAQAMLSGEPTIIEASAALLKAIVTRNPKAMIRLYSTGAFYFALAYPGSNLLSIAQLFSVTHVHQAFHGGEEAAVSSSLPLAKRSVLGGLLPESLLYVLERSGPVAFAAAMVSDSDTPEIIWTHKMRAENLIRQVLQHLGDFPQKLSQHCHSLYDYAPMPPVTYPELKDEMWCHRYYLRNLCDEIRFPNWPIVEHVEFLQSLLVMWREELTRRPMDLSEEEACKILEISIEEVSRDDAPKKASFESIEEISSISKQIEYIDEEKLKRQYRKLAMKYHPDKNPEGREKFLAVQKAYECLQVSMQGLQGPQTWRSLLLLKGQCILYRRYGDVLMPFKYAGYPMLLNAITVDKDDNNFLSSDRAPLLVAASELVWLTCKSSSLNGEELVRDGGIPLLATLLSRCMCVVQPTTPATEPSATIVANIMRTFSVISQFESARTEMLEFAGLIDDIVHCTELELVDAAIDAALQTIAHVSISSEFQNALLRAGVLWYLIPLLLQYDSTAEESDKTDAHGVGTSVQIAKNSHSVQASRALSRLSGLGDSETPIPYNQAAADALRALLTPKLASMLKDKLPKDLLSTLNSNLESPEIIWNSSTRAELLNFVEEQRARLSHDGSCDLKDSHYFVYEALSKELYIGNVYLRVYNDQPDFEITEPDNFCLALVDFISHLVHDEPAANVNVHVNGDVTTESSADSSDDSSAPVDGKATDRKEFELIKNLQYGLISLQHLLTKNPNLASVVSTKEKLLPLFGCFSLPVTSASNIPQLCLIVLSRLTTYAPCLEAMVADSSGLLILLQMLHSSPSCREGALHVLYALASTPELAWAVAKHGGVVFILEVLLPIQEEIPLQQRAAAASLLGKLVGQTMHGPRVAITLARFLPDGLVSVIRDGPGEAVVSALEQTTETPELVWTPAMAASLSAQIATMASDLYREQVKGSIVDWDVPEQASAQQEMRDEPQVGGIYVRLFLKDPKFPLRNPKRFLEGLLDQYLTSIAATHYDGQAVDTELPLLLSAALVSLLRVYPALADHVGYLGYVPKLVSAVAYEASRETMASEAYATEDASLQQNSQTPQERVRLSCLRVLHQLAGSTTCAEAMAATSVGAPQVVPLLMKAIGWQGGSILALETLKRVVVAGNRARDALVAQGLKVGLVEVLLGLLDWRAGGRNGLFSQMNWNESEASIGRVLAIEVLHAFATEGAYCSKVRDILNASDVWNAYKDQRHDLFLPSNAQTSAAGVVGLIESSSSRLTYALTAPPPQGSQTKSPTTAT* |
| evm.model.group2.1094 | MVKRSKARVSEEHIEEEDEEMKEEEFDNGSTSSNEKSLYEILGVDKGASQQEIKKAYHKLALRLHPDKNPNDEEAKEKFQQLQKVISILSDEEKRAVYDQTGCVDDADLAGDVIQNLQTFFRAMYKKITEADIEEFEASYRGSDSEKNDLLELYKKLKGNMDKLFCCMLCSDPMLDSHRFKDIIDEAISAGDLKTTKAYEKWRKKVSKTKPPTSPLRHRKKSKKNSEDLYAIIAQRQNERRGKIDSMFSSLVQKYGGGEAAPEPSEEEFEAARRKLENRRTSKRK* |
| evm.model.group2.1135 | MNKVSRIAATINSQSNPFQLRASHFHCTPVLERRRRTHWNFESSFRSSPRKFNQYTKRLRKQSLLRNVNEFAEHLFQSWQSDKDEYDQPPRQGNSWFKPSFREDGYKKGKSRYRTSQACRRDFEFCNDNYDDVEFETIFRSAFGGDRYFYWSFTTDDGPRHRNSSSYSNSRTSNGWKYHYDEEYDSSSEYEKPMTDMTSDRLALGLSASGPLNLDDVKNAYRACALKWHPDRHQGSSKVIAEEKFKVCSVAYQSLCDKLALN* |
| evm.model.group2.1334 | MKNRLALTLFVFGLLFLLFVECKKLDPYKVLGVDRNASQREIQKAFHKLSLQYHPDKNRNKGAQEKFAEINNAYDILSDEQKRKNYDLYGDEKGSPGFDAGDPGEHGGYTYFTSGGPGQSGFSFRPGEWQNMGGREGSKSFSFSFGGPGFGKQSSFGFGDIFSNFFGGGMGGGSQFGGFGSTGRSQYGSRHSAGSIPSVNSQLYRKEIVDKGMTWLLLVYTPNLQGIQYYESVIEEVATSLQGALKVGSINCGSDASFCKELGVYPHRQPKIFVYSYASSEKGSMVEYDGELDVKSLKSFCQDHLPKFSKRINLDNLNAVSEAGRLPKVLLLSTKKNTPVIWRALSGLYRKRFIFYNAEVRDVSDPSVRNLGIDSLPAVIGWLSNGQKQILRSGISVKDLKSAIKELSGLLDNFEKKNKNAASAKKDDTESGDKSIPLLTASNFHDICGDKVPICLVGVFRSSTTRDKLQKILQSVSQKSFSRRQSVTSNSRDQVSYALLDASKQQSFLNALDKSGFKSADKLLVAYKRKRGTYATSKSEITEEAVESFISSVLNGDVQFTKARQKPTLK* |
| evm.model.scaffold291.46 | MILSNNLGRKTHYEIIGVKEDASQEEIRRTYRSAILNFHPDKLQKSSETSSPEHEMSNQFLEVQKAWEILGDPRSRAFYDNELRTLRQDVITADDVSLEDMVIEEDDGSCFELSYHCRCGDFFSIDSSELAEMGYPFSRNGSEISLEAPSTLPASVVLPCGSCSLKIRLVIDGHVKLVSELNN* |
| evm.model.group0.2150 | MSPAFVDSGAPPPCNSQVFSSHFNSSHNIDKNSIAFIGFSNHMDPNFGFSSPSLGPSRTATGLSRPPRLAKIRKPLAGHRPNLSRPVSQLGAGRRLEDSGLESARVDPGFSTAKPAFEPSQQNVDRGFVFGSNDSGHPISSSGKSGVFSEHVANNNVEASDVLDERRRLRIDPSFSTAKPTFEASQHNAGLGFEFGSNDGSHTFSSSKSDLFSEDVANNNAYASKIVDEMRRLRIESQRGYSSSNFASGTSGNIHLSGKDHSLQGVDESVVSELPDEMRRLYIESEHFGKLYGGNIEELPNKMKKLNVKDYEHDDLKNLGRSNSKSCVGSLDNVLPNKMQNLKIEDSLYGSVNEKVDDFSSGDKNKFMFKDSGNANEPVDLNISSAAQNSSNHLKTKTSLSSGAETMHDVQAKSLGTGNWHNIAGSFNSGFSFQAGLESRNSGSHVSFKHESNSTSLPAFASSGIHFRPVRTVPEMPPADTGDKKFEFSFTSKLDSMAARPVEFKTPDPKAHSLFGLNRKAETKRESAKDGALKKKKGKWKKSAQVPLMFQQDFVFKENLQENAESSEQYSPMDLSPYEETLANNSFSRETSVASEESFHLDENNSSSEAYRNVSNDIVDEVLVSATEGLHINECDIKGNEGQGEESAYCLNEGSRMESPEEDAVSGAETESFKSATDELDYSTDSFVTAADTEVSSNSKLERQDSDSGTQLKHETNLADMVQSSFTFATSSFSLSKSSALMRNQKKKSQMKLCQDSLSHASSHLPSIQVSESSLSLPEQHQKGSFSTVFSQKRDKLEQVKELVTKQDSPTAVSIAAQESCEKWRLRGNQAYAKGDFSKAEDCYTQGVNCISQNETSRSCLRALMLCYSNRAATRMSLGRLREALEDCTRASAIDPNFLKAQVRAASCYLGLGEVEDASLHFMKCLQAGPDVCADRKLLVEASEGLEKAKKVAECMKHAAELLGRSTSSDIDSAVSVISEGLMISSYSEKLLQMKVDALLTLKKYEELIQLCEQILGYVESNFSTSGVGSHSAAFHGSDLKRAPSFKVWSCSLILKSYFYLGRLEESLVFVKKQEESASLVERSESRTLESMIPLIGTIREMLHHKAAGNEAYKSGKHAEAVEYYTTAISCSVESRPFAAICFCNRATAYRAMGQILDAIADCCIAIALDGNYYKAISRRAALYEMIRDYGQAVADFQKLVSLLTKEVDKKTNHSGTSDKMDYMNELRQARLKLSEMEEAARNDIPLNMYLILGVDPSAAASEIKKAYRKAALKYHPDKAGQSLVRNENPDDGIWKEIAEEVHKDADRLFKMIGEAYAVLSDPTKRSQYDLEEEMRNAANRGNSGKTSKTSSDFHNYGNRRHWQEFRRSYGNSTRGSERNHNWYS* |
| evm.model.group0.1338 | MGGSKMEGPSVPVLRRDPYEVLCVSRDSSDQEIKTAYRKLALKYHPDKNADNPEASELFKEVAYSYSILSDPEKRRQYDNAGFEALDAEGMDMEIDLSNLGTVNTMFAALFSKLGVPIKTTVSANVLEEALNGTVTVRPLPIGTSVSGKVEKQCAHFFGVSITDEQAQAGIVVRVSSAAQSKFKLLYFEQDTNGGYGLALQLAMAKDPEAAFFKRLEGLQPCEASELIAGTHIFAVYGDNFFKPATYTIEAICAKSYEDTTQKLKDIEAQILRKRNELRQFETEYRKVLAQFQEVTSRYGQEKQSVDELLKQRDSIHSSFTYSKNLLLFLVGVDI* |
| evm.model.group0.1914 | MRWDGLCDFEQAENEESGDHSDVNFDFLSVLSKPKDYYKILEVDYDASEEVIRSNYIRLALKWHPDKQKGKDNATSKFQEINEAYQVLSDPVKRHEYDKQGMFYSYDSNIMDYLNRYKGLILTCNGLGMKFSIW* |
| evm.model.group0.1822 | MEIVPDRSSSYYSVLGICRHASDAEIRGAYRKLALKWHPDRWMKDPAIAGEAKNKFQQIQEAYSVLSDKGKRTIYDAGFLEDLPEEDDDDKEFDKFMREMISLMESEKSQEERSLEDLQRLLMEMMDRDERLKFELDLKQSHGGAPKRRRMNTSGLDDP* |
| evm.model.group0.2339 | MFGRAPKKSDNTRYYEILGVPKTASPDDLKKAYKKAAIKNHPDKGGDPEKFKELAQAYEVLSDPEKREIYDQYGEDALKEGMGGGGGMHDPFDIFSSFFGGSPFGGGGSSRGRRQRRGEDVVHPLKVSLEDLYLGTTKKLSLSRNVLCAKCNGKGSKSGASMKCSGCQGTGMKVTIRQLGPGMIQQMQHPCNECKGTGETISDRDRCPQCKGEKVVQEKKVLEVRVEKGMQNGQKITFPGEADEAPDTVTGDIVFVLQQKEHPKFKRKGDDLFVEHTLSLTEALCGFQFIISHLDGRQLLIKSQPGEVVKPDSFKATNDEGMPMYQRPIMKGKLYIHFNVDFPDSLSPDQVDALAKILPPKPQSQLTDMELDECEETTLHDVNIEEEMRRKQAQQQEAYDEDEDMHGGAQRVQCAQQ* |
| evm.model.group0.2332 | MGDNSGTTSSATAAAAASTANEDLLLKKFFAEVSEVERDNEVNRILSCFKLNAFEYLNLPLDSSVDDVKRQYRKLSLLVHPDKCKHPQAKEAFGALAKAQQLLLEPQERDYLLNQISAAKEELRAKRKKQLKKDTATKLKSLVDEGKYEQEYERSEEFQQQLKLKVRELLTEQEWRRRKMQMRISEEEGRLKKDEEETKEMWKRKREHEEQWEGTREQRVSSWRDFMKGGKKVKKGELRPPKLKTEDPNKSYVQRPLKRG* |
| evm.model.group16.800 | MEHPFFPTTTTRAEALRWLTIAEKLLSARDLMGSKSFASRARDSDPTLSPADQILAVADTLLAGDRRIGNNQQDWYAILRLTPQQGRDSELIAAQYRNLALILNPQKNKFPFAEQAFRLVVDAWSVLSNPSRKSMYDKELAFYLNPQQQQPDPFTNPIPAMHQNFIFFGGSSSSGVTHPPQPQGALHFSQAQVHSSSQVDTVSGGSGSAREPQNFMSFTSGSNLVFGSGSGSGSGSGSGSGSRAGQETLNKQVQENYSNFVGNNVATDAPGTVQEQVNDKHEQPQQNYSNVGDKTSHNVNVSENEGERIEEEEDMEGMEEERVDAPVNNDVSTIWTACPYCYYMYEYPRMYADCTLRCQNCKKAFQAVVIPSPPPIVDGQEGYFCCWGFLPLGFSMENWEKNKGAASSWTPFSPMFTCPQAGNNGAKKGGRKASAPRVYFDDDEIFVEISDESESDDDWRNDREKKKKKAKNAKGKGASGATTRSTKKAQVDKGKNVQDGFVSPDGD* |
| evm.model.group16.75 | MVRCNGIRLARRSLSSFYRNSSPSSGGCRSFGNALCNQSRILFSSNFGNVGSEKYWLKLGLFNDNLGTARSIHGTAQLSRDYYNVLGVSKNATASEIKKAYYGLAKKLHPDTNKDDPEAEKKFQEVQKAYEVLKDEEKRQQYDQLGHEGFESASNGEGPGFNPFEAGFNPFQDIFKNADIFNIFNRDMGGEDVKVSVDLSFMEAVHGCTKTLSILTDMACDTCGGSGVPPGTRPETCKRCRGSGMVISQNGPFTLQSTCPNCGGAGKIVSSFCKSCRGKRVVRGSKTVKVNIMPGVDNNETMKIPRSGGADPDGNHPGDLYVMIKVREDPVFRREGADIHVDAVVSISQAILGGTIQVPTLTGDVVVKVRPGTQPGQKVVLRKKGIKVRNSFSFGDEYVHFTVSIPTNLTQRQRQLIEEFAKEEQGEQGEDDKGAAASASG* |
| evm.model.group16.670 | MDREGGSGVGSLCYYSVLGIRKDASFSDIRSAYRKLALKWHPDRWARNPSAAGEAKRRFQKIQEAYSVLSDKGKRSMYDAGFLDLFEEDEGMGDFLHDLMNMMEQNVGAEEESIEDLQKTFVELFGEDLKDMMGESDDPAIRKRARDGGSNTRGTSKRNSRT* |
| evm.model.group8.197 | MVAIKGLLLCSKEHGKLLKGQDRSFQGAALADGLLWVCKMWERSIVLVGLLEKLFSHGLPFLYWAWLNGAASYYVELLPKLDINGAAGAAVQYLGYTPGLFIVGLFAILILWMYANFWITGALFIVGGYLFSLNHARLVVFMATLYAIYCVKVRVGWLGVFLSINLAFLSNDILNYSMKWCDNWSENTHFEEHKEPESFIGDDFSTECGYSAPTEEENLHSCKSASKPASTSSFVEKPKESVAKQVVREDINSITEMKRILGSCNHYEALGFPCQRKIDLVLLKKEYHKKAMLVHPDKNMGSPLASESFKKLQCAYEVLSDAVKKRDYDEQLRKEESKSVLQRSASTSDQNTTDFCSEESRRIQCTKCGNSHIWVCTNRTKAKARWCQDCCQYHQAKDGDGWVEYKGSSVFDRSQQVEIPCAFVCAENRIFDVSEWALCQGMACRPNTHRPSFHVNMVGLEKSTQRSNSSRYPWDLDAEMMDEEEEFELWLQQALASGLFCETSKRRKTWSPFKLPQKKVMKQWRRFS* |
| evm.model.group8.552 | MGVDYYKILKVDKNANDDDLKKAYRKLAMKWHPDKNPNNKKEAEAKFKEISEAYEVLSDPQKRSIYDHYGEEGLKGQVPPPDTGGPGGATFFQTGPNVFRFNPKNANDIFADFFGFSSPFGGMGGGSGTRGGSMFSSSMFGDDIFSSFGDSRPMSSGPRKAPPIEQMLPCSLEDLYKGTTKRMKISREIADASGKTLPVEEILTIDIKPGWKKGTKITFPEKGNEEPNVIPADLVFIIDEKPHGIFSRDGNDLVVTREISLAEALAGYTVYLTTLDGRNLTIPINSVIQPSNEEVVPREGMPIPKDPTRRGNLRIKFNIKFPTRLTAEQKSGIKKLLPP* |
| evm.model.group8.797 | MESNIEEALRAKAFAEKQFVEKDFVGAKNYALKAQKMCPELEGISQMVATFGVYIASEAKINGELDFYSILGVDPSVDKSKLKKQYKKMAVLLHPDKNRTVGADGAFRLVSEAWTLLSDGVKRSSYDQRRNLFAGYSAGDWWL* |
| evm.model.group8.158 | MLGTLILPSQPSFASQFRPRRMFVCSAVAEVVPAAAGVDRKSLYEVLRVQRNASQMEIKTAYRTLAKLYHPDATSRFMDSPIASSTDGRDFIEIHNAYATLSDPDARADYDFSLNINSQRRWNTAGLSRFRSPGRGRFYPSRRWETDQCW* |
| evm.model.group14.46 | MARKRNQQKNGLDKQLPNHKKGGSDSGPSPLANEDRGNLNDEEVGLGGELPNGSHSISSTKNINDMDHVEDGKKSKKKSRKSQRKERKVANETVKDHAVPCNDNTEIDKSNTSTDEALNMRDESDLPRIPISDNMDMLGNSSNALHRDEGIKDAEFPETVVFKFLRTAALSVARSFADWVERHKPTFATLKSNTLKARDHVRMKMQHAQPIILWWITHIGNIMLLLFMVWLECTLRGIDSFLRMGTTSFFSILWCSVLSVIAMVGISKFLFALAVVAAVGLFLGFTLAVVLTGIVGIIFLWFYGSFWTTGLIIFLGGLAFITTHDRIALLLASVYSIYCAWNYVGWLGLVFGLNLSFISSDALLFLLRNIIKEQRAPNSPPEQAAGVQGESFFFHNESTHASTTDTGATGVRADRSPGVPSTSGSDSETTSEDEVVRLLNCTDHYAALGLPRFENIDVSVIKREYRKKAMLVHPDKNMGNEKAAEAFKKLQNAYEVLLDSFKRKEYDDQLRREELLNFFHKYKNTSHQNQGLNLFKSGFARPDADSEDPLGLSRRIACKKCGFFHLWVYTKKSKSKARWCQECKDFHQAKDGDGWVEQTSQPLFFGILQQVEAPSAYVCAGGNVYDVTEWYICQGMRCAVNTHKPSFHVNTNVVSKNAHAKGPSSSGQRGGGIPTPNIEETMTEEEFFEWLQNAMQNGMFEDFVGSSSGSQSPSGFNPKSSGSNSSSKRKKKGKKQW* |
| evm.model.group14.178 | MLILGMERNCSVDDIKTAYRKLSLKVHPDKNKTPGAEEAFKKVSVAFKCLSDNESRKQYDQMGSGNEFEHHYQQDPFRRRRRRTWNEYFNDDVEPEEFHRQFFGHSYAFREAHFYRTRTNHAPAHQRKDSHNGLPNTIWWLPLLIFILFASFMFMVPEYSSNRGFSHRIRKTTLNYGVDYFVKSPQFDMKYPMGSPSRTQIENNVINEHRLKLERACDRETSWIPPHRWHYTPRPHCEILESFMA* |
| evm.model.group17.100 | MPGHKSKAEKGDAEKQLRRDPYEVLGVNRNSTDQEIKSTYRKLALKYHPDKNANDTTAADMFKEVTFSYNILSDPDKRRQYDAAGFEAVESESQELELDLSSLGTVNTMFAAIFSKLGVPIKTTVSATVLEEALNGAVTIRALSLGQPLYRKVEKQCAHFYSVTITEKEAQAGLVCRVYSPDKSKFKLLYFDQEDNGGLSLALQEDSSKTGKFTSAGMYFLGFPVYRLDPTHNSTAAVKDPDAAFFKKLDGFQPCEITELKAGTHYFAVYGDNFFKSVTYTIEALSTAPFPEEKENLRSVEAQILSKRVELSKFETEYREVLTQFTEMTSRYAQEMQAIDELLKQRNEIHASYTTTPQIKRSSSRNKNKAISKENEDGLGKENKHSRDRSKKKKWYNLHLKVDKRKPCGME* |
| evm.model.group17.397 | MECNRDEAMRAKDIAERKFLAKDIKGAKKFAVKAKNLYPELEGISQMLMTLEVYISAEEEKIHGESNWYGVLGVTPLADDETIRKQYRKLALLLHPDKNRSIGAEGAFQLVSQAWSLLSDKSKRVAYEQRYRAASQPKKKTTKEGPSPSPMQNGFYNFANTAASQTRVPKGNISKKNPSSGPYPSRKKERQTFWTVCHRCKMQYEYLRMYLNHNLLCPNCHEAYFAVEIAPPSTKVSKISSQTSNSQQRKNSSQQGADATRNNSAAQHGTPGSSKDQANFQWVPFHESAGAQSSVQAANMVHQAYEKVKRERQKAQAAARREEALRRKNLAPKRTMGAESSGPSDAAKRRKGVIDCGINKEKTQQVNCESGTLHRTNISGLKQDIFQKYRRKIDGAYHVDIKQMLIEKAREEIYKKINGCISDSMVRNAAGEEVSENTKENETSNVKDGSVTHGAISNQRKLCESVEAESIFPLGKLNIGSLGPYVDSELVEQMSVNVSNPDFHDFDEDKTEKCFGDNQVWAVYDDDDGMPRHYALIHNVISLDPLKVRMSWLCSVTHSKQGHISWFLSGFSKTCGDFEIGRLETCSTIDCFSHNVRWMKRTSKTIQIFPRKGDVWAMYRNWSPEWNELTEDEVIHKYDMVEVLEDYDEELGVIIIPLVKVAGFKSVFHQHFNSMEIQRIPKEEMSRFSHQVPSHLLTGQEGSKSPKGCLELDPAAIPLELLQVKSNVEEIELIKIDDDDDIEFVKVVDCNRNAHANMVEGIS* |
| evm.model.scaffold425.3 | MAHRRSKLLLFFCFISYYVIAIAAKSYYDTLQVPKGASDEQIKRAYRKLALKYHPDKNQGNEEANKRFAEINNAYEVLSDSEKRGIYDRYGEEGLKQHAANGGRGGGGMNIQDIFSSFFGGDPTEEEEKVVKGDDVIVDLDATLEDLYMGGTLKVWRVKNVIKPAPGKRRCNCRNEVYHRQIGPGMFQQMTEQVCEQCPNVKYEREGDFITVDIEKGMHDGQEVVFYEEGEPIVDGEAGDLKFRIRTAPHDRFRREGNDLHTTVTITLVQALVGFEKTIKHLDEHLVDISSKGITKPKEVRKFKGEGMPLHFSNKKGDLYVTFEVLFPSSLTEDQKTKIKEILG* |
| evm.model.group1.2318 | MVAIKGLLFCSKNCNELFEGQDRNFHGAALADGLLWVCEIWENSAVLVGFLEKLFPFWIPVIYWAGISGSAYYYVELLPKLDINGAAGVAVQFLGYTPGFFIVGLFGILILWMYANFWITGALFIVGGYLFSLSHARLVVFMATLYAMHCVKVRVGWLGVFLAVNLAFLSNDVLNYLIKWCDNLSESTHFEEHKESDSFTESDFCTDCEYSTPTAVEGEAEEKLHSCKSASKLATPSFVEKPKECAAKKVVKEDVTSIIEMERILNSGNHYEALGFPRQKKIDFVLLKKEYRKKAMLVHPDKNMGSPLASESFKKLQCAYEVLSDALKKRDYDEQLRKEEAKCVMQKSASTSYQNTTDFCSEESRRIQCTKCGNSHIWVCTNRTKAKARWCQDCCQYHQAKDGDGWVEYKGSLVFDRPQKVEIPRAFVCAESKIFDVSEWAICQGMACRPNTHRPSFHVNMVGLEKSPQRSNSTRYPWDLDAEMMDEEDDFEMWLQQALASGLFCETSKRRKSWSPFKLPQKKGKKQWRRSS* |
| evm.model.group1.471 | MEPSAKIRWWAPTIILVVSLLVQPSVSIYCDEDDCYDLLGITQSANASEIKKAYYKLSLKYHPDKNPDPESRKLFVKIANAYEILKDEATREQYDYAIAHPEEVFYNTAQYYHAYYGHKTDTRAVLVGLLLVLSGFQYLNQWTRYKQAVDMVKKTPAYKNKLKAMELERTGGMTSKKKSNKQMNKKMEEDFSKELEPQIKGAEKPSVWELVGVRFILLPYTLGKLLLWYGCWFWRYQVKKSPYFWYDAAYLTRRSLGVPLDSWRYIDESTKDDLVQRRLWEKPNLESYLAEMRKESKRRR* |
| evm.model.group1.1390 | MGSNMEEAVKAKILAEKQFVEKNFVGARNYAVKAQKLCPELEGISQMVATFGVYMASEAKINGELDFYSILGLDPSADKSKLKKQYKKMAVLLHPDKNKTVGADGAFRLVSEAWTLLSDSAKRSSYDQRRTLFAPYSTGAGGYENCSKFSSSHGRLDTFWTVCTSCHVQYEYLRKYVNKRLSCKNCRGVFIAVETGLAPVNGSFPYTTYPFAAENGYGSHGCGVTYMSTTPGYCAPNGASGHRTGYRSEYVSNISFQDSTSGNSVGALDPKGSSVSPFVFYQASGDANKTKVNGKHHAGTPTGHVGSNGYTASNEVLKTRRGRPAKKRKVELGSSYANGHEEICSNIVLEQKISNGNETNKPASRLSSTFESLTRRCSAAPAIDGRQLLIEKTRSEILRKLEELRLASEAEAAEAEMRNAINKQPELKRTVSMSITVPDPDFHDFDQDRSEECFKPKQIWALYDEEDGMPRLYCLIREVISVKPFKIFISYLSSRSDSEFGSVNWLDSGFTKSCGSFRVFHSETVEQVNIFSHLLSREKAGRGGCVRIYPRCGDIWAIYRNWSPDWNRATPDEVRHQYEMVEILEDYSEEKGVRIIPVIKVDGFKTVYQRNTNKDAIRWIPRREMLRFSHQVPSCPLKVEGTNLPEGCWDLDPAATPEELLQVETELHNSTSPHCIEKTSRTPEEQYTAEPIGQPEKKLSQSDICASTPDKGPQVAGGACVEERTSVELPRGQEGIPVEVNVE* |
| evm.model.group13.908 | MAAAAAAAAGATATATSMICPSSLSFTPPSQSNLCPSMGSSSSFFFGGGAAIWGNKKCLQVSLSSDTQLDKKMSARRFGRGVVVAAADYYSTLGVPKSATSKDIKAAYRKLARQYHPDVNKEPGATEKFKEISAAYEVLSDDNKRAMYDRYGEAGVKSTVGGAGGYTATNPFDLFEAFFGPSMGGFSGMDGSGFGTSRRSTVSKGEDLRYDMRLEFSAAIFGTEKEFELSHLEVCGVCAGTGAKVGSKMRICSTCGGRGQVMRTEQTPFGMFSQVSVCPSCGGDGEIISESCRKCSGAGRIRVRKDIKVKIPPGVSKGSILRVAGEGDAGPRGSGERLSFEFKRNDEVF* |
| evm.model.group13.333 | MASSEKRCLYEVLGLSRECTADEIRSAYKRLALQRHPDKLVQSGVSEAEATAAFQELVNAYEVLSDPKERAWYDSHRNQILFSSTSAASGSSSFVPDLFSFFSNSVYSGYSETGRGFYKVYGDLFEKIYANELNFAKKLGLPLPKEAPVMGNLQSPYVQVTAFYNYWMGFITVLDFFWVDEYDVMAGPNRKSRRLMEEENKKLRKKARREYNETVRGLAEFVKKRDKRVIDMQIKRNEEMERRREEEKQRKKELERQKAEKARNYEEPQWAKVEEMDDDDEPEDLVEEKKKELYCVVCSKKFKSDKQWKNHEQSKKHKEKVAELREAFSEEDKDYKATGRNENEAELDKVVDGNGEGYFSADYSVNELQDQFEGSVGIRGDDSDDGKTVSSESNEFADVDNHSGEKGIAAELGSDDDEASFLGAMLSGRKSRNKVGSQPKAPAKKIQVEVDSDELEFMDYNDTKASRRNRGGRRQRGKRQEEKAETGRADVAERNGEAEEHDERDDDVGESTSHPLPESEARIADDATEKGHKHPQHTVNKKSTTKKENASKSKFASKGRKQKAAASGSDNICEKCGEQFESRNRLHKHLGDTGHAALKSR* |
| evm.model.group13.44 | MQVPQIPNFCSINSNAINPKFVFCNSNYLSPPNSKTLGFSGTVFPTLKAISRIPSWNNFNNSTRRCRFAPFIAAAAAKRASGSDYYSVLNISRDATLQEIKAAYRLLARKYHPDMNKGPGAEEKFKEIGAAYETLRKGLYMTALARKV* |
| evm.model.group13.964 | MAAALGCGSGSWVQLKDNLSRKKAGKNNKKGYRVSCVSSSALSDPYKTLRIQPGASESEVKKAFRKLALQYHPDVCRGSNCGVQFHQINEAYDIVMSNLRGERNTPRVETYGEDYFADEPLRGMYDQSWELWEEWMGWEGAGIRDYSSHINPYI* |
| evm.model.group13.704 | MAAEEENSNDFYNVLGLKKECTTAELRNAYKKLAMKWHPDRCSACGNLKYLEEAKKKFQAIQQAYSVLSDANKRFFYDVGIYDSNDDDDKNGMGEFLNEMTILMSQNKSNENRNESFEELQELFEEIFHSDDTEDFGSSSRFVMTPPTHLSSQDSCNETYSISNKRNSSSANFSIGATGFCIGASGTPGERTQRRTRGSRRDGRKQKTSSGKDVSSNKYSTVSA* |
| evm.model.scaffold246.24 | MTTISSLYDVLGISTSADEHEIKAAYRRLAKKCHPDAVAMAKNYKSTNEFLKIHAAYSTLSDPHKRATYDRDLLRHRGSYNSSVVLDSSTLRWSRYPSYGVRNWESDQCW* |
| evm.model.group5.587 | MECNKDEALRAKSIAEGKFEQNDFIGAKTFALKAHNLYPGLDGITHMLSTLDVFISTANKINGEANWYGVLDVDPAADDEAIRKQYKKMALMVHPDKNKFTAADGAFKLVSESWSLLSNKEKRLAYDQRRAFKGFQQSFPTYGGGPSVPSTENQGFKHEDRSFYHSHGPSASRENWFFRHTDGPSATSKDNQFYNSHVPSFENPFFKHASGPSASSPSLDNLFYNFAGWHTPASQPKRTSASKSRKRSTNVPSRSAPTLSCQRPGSFWTVCCWCNMHYEYHVMYLHRTLQCPSCRHPFMALEVASPHKDSGWFVHGKQP* |
| evm.model.group5.1544 | MAIIHCGSTPVTCWGVQPQFIVRSSVTNKLSTYLHCTSRTSLASQNATFFSQRSLNAFLSPGSYVNSCRRRGARLVVRADRDYYSVLGVSRNASKSEIKSAYRKLARSYHPDVNKEPGAEQKFKEISNAYEVLSDEEKRSIYDQYGEAGLKGGGTGMGDFSDAYDIFESLFGGMGSMGGMGMGGRRSRNRATEGEDQVYNLVLNFKEAVFGIEKEIEITRLESCSTCDGLGAKPGTKSSKCSTCGGQGQVLSSARTPLGVFQQVATCSVCGGAGEISTPCNTCNGDGRARKSKRISLKVPAGVDSGSRLRVRSEGNAGRRGGPPGDLFVTIDVLPDPVLKRDDANILYTCKVSYIDAILGTTVKVPTVDGMVDLKIPSGTQPGTTLVMAKKGVPYLNRSNMRGDQLVKVQVEIPKRISNEERKLVEELANLNKAKASSRRK* |
| evm.model.group5.913 | MEHHTTTSRAEAERLLGIAEKLLRNKDYSGSRDFAILAQETEPLLEGSDQILAVAEVLLAAENKRLNNNQPDWYSILQIPQRTDEAELIKKQYRRLALLLHPDKNKFPFSDSAFRLVADSWAVLSDPAKKSVYDNEFSARFTKVDLVAMKKQQQQRKQQQQREENSQKLPVRRSARGGSSGATPTPTPIPTDGGVKVNNKRNISGNSGWSSGNFWTACPYCYNLYEYSRVYEGCCLRCDNENCKRAFTATEILSMPPLVPGKEAYYCCWGFFPMGFVAGGSGFPKWMPPMFENSGPAVVEEEHPIAPPRNATPGAAATSSAKKKRGRPKKNV* |
| evm.model.group5.275 | MVKETEYYDVLGVSPTATEAEIKKAYYVKARQVHPDKNPNDPLAAQNFQILGEAYQVLSDPTQRQAYDAYGKSGISTESIIDPAAIFAMLFGSELFEEYIGQLAMASMASLDVFAEGEDFDTKKLQEKMRTVQREREEKLAGILKDRLNLYVQGNKEDFIRHAEAEVSRLSNAAYGVDMLSTIGYIYARQAAKELGKKVMYLGVPFIAEWFRNKGHFIKSQVTAATGAIALIQLQEDMKKQLSAEGNYTEEELEEYMQSHKKLMIDSLWKLNVADIEATLSRVCQMVVLQDNNAKKEELRLRAKGLKTLGKIFQRVKSVNGNDTETMMNDSSNKLRGSETSNNPAPSSTSARSLNEEVSYNAPAAQSPYVEAPQFPGAQFDNFPMPTAPPGAHKQQ* |
| evm.model.group5.504 | MFGRAPKKSDNTKYYEILGVSKNASQDDLKKAYRKSAIKNHPDKGGDPEKFKEIAQAYEVLSDPEKREIYDQFGEDALKEGMGGGGGGGHNPFDIFESFFGGSPFGGGGSSRGRRQRRGEDVVHPLKVSLEDLYNGTSKKLSLSRNVLCPRCKGKGSKSGASMKCAGCQGSGMKVSIRHLGPSMIQQMQHPCNECKGTGETINDRDRCPQCKGEKVTQEKKVLEVVVEKGMQHGQKITFPGEADEAPDTTTGDIVFVLQQKEHPKFKRKDDDLFVEHTLSLTEALCGFQFILTHLDNRQLLVKSEPGEVIKPDQFKAINDEGMPIYQRPFMKGKLYIQFTVDFPESLIPEQCKALEAVLPPRTTTQLTDMELDECEETTLHDVNMEEEMRRKQQQAQEAYDEDDDMHGGAPRVQCAQQ* |
| evm.model.group5.1476 | MVKETEYYDVLGVSPSASEEEIRKAYYLKARQVHPDKNPNDPQAAERFQVLGEAYQVLSDPLQRDTYDRNGKHCISKETMLDPTAVFALLFGSELFEDYIGHLAVASMASTELAGESDNPEKVHDRLKAVQREREEKLARNLRDFLHQYVRGDKQGFFKQAESEAQRLSHAAFGAEILQTIGYIYTRQAALQLGKKAIYLGVPFVAEWVRNKGHFWKVTVHCSKSMSTAGAFQLLQLQEDVRHQFKMDGSGPENDVESHLRLNRDTLMNSLWKLNVVDIEITLLHVCQMVLQENCIKKEELKLRAVALKILGKFFQVV* |
| evm.model.group5.968.1 | MVNGEDDKRSWDNRWPRANSEKEEEMRLWGILVFGLIGATVTTFAVTQLRSTVDRIYSQLSRSQSSWKNGSYFRTSFQEEARKRYNRRMREEYEAEMERLERIRRMQSVFNRERNKFKKSYDNWQQYGPDAHHQHFQRNDWYWKEDTSFRDHSSRFRETSSAGASAPLSHHYSVLGLDRLRTRPYTDDEIKSAFRAKAKQFHPDQNQDNKEFAEAKFKEVMTSYEAIKSERKNNGHH* |
| evm.model.group10.186 | MGTHNRQSGQVETKPETRRFLTAAEYFLRHRNFADCRKYAVRARDSDPTRPGPTQVLTIASVLSAPNIFSVSHDYYSICNLPHFESDAARIESSFKSLASILSPNSNPYPHSSEAFDVVMQAWTVLSDPIEKAKFDDELRRNMGGCMSGIDGGTFWTICPYCYHVYEYEKVFEDCYLRCPNEGCRRVLHAVAIEAPPPREVVEKGYYRCAGFMPFVIRSSNGEEVGQNFWSPFGPPIGSASKVRDHKSDSSGDGLVIDISGDEDEGTENAETVGEMGFQDKMKIAAEANGGRMESTGGHESGFMQNRVNEVRMRRKKSVPWNSKKLMGRGFVIDKNQAQSIYTDGEEGSFNVDANEGENLELEFGGKTGNAFETGVEFFDGEDDVLVGLQCDFDLGNGDYEFINANNGTL* |
| evm.model.scaffold69.59 | MFGRALRRSNNLKYYEVLGVPRSASQDELKKAYKKAAIKNHPDKGGDPEKFKEVAHAYEVLSDPEKRQIYDEYGEDALKEGMGGSSHTHDPFHIFESFFGGGFSGGFGGGFPGGFGGGFSGFGGGGSRSRKRQGEDVVHPLRVSLEDLYNGTTRNLSISRNILCTKCKGKGSKSGNLGRCFGCQGTGVRTTSRQILPGMTQRIQHVCSECRGSGESIRERDKCSQCKGMKVTQEKKVLEVHVEKGMQHNEKIVFSGEADQAPDTITGDIVVMLQQREHPKFKRNSDNLHVEHNLNLREALCGFQFVLTHLDGRKLLIKSKPGEVFKPDCYKTINDEGMPHYKRPFIRGQLIIHFNVEFLDSGFLSPEKSQILRTVLPVKSDKHLSRKVLARCEETTLIDVDIEEEMRQKEQRRQREAYDEDDDATVHQVACNQQ* |
| evm.model.group6.489 | MGVDYYNILKVNRNASDEDLKKSYRRLAMIWHPDKNANKQEAEAKFKQISEAYDVLSDPQKRQIYDLYGEEGLKAGQFPPPPRSSRAQSQGFYYNNQQHPNPNFRFNPRNADDIYAEIFGENSNGNAGGNASSSSNSGGTRRDGYFRSTAMNGGGGREYSGGGGSRKAAPVENVLMCSLEELYTGSTRKMKISRSVLDSHGRTRVLEEILTIDIKPGWKKGTKITFPEKGNEEPGIIPADLIFVVDEKPHPLYTRDGNDLVVKQEITLLESLTGKTFELTTLDGRSITVPLTEIVKPGHEIAIPNEGMPVSKDPRKKGNLRIQIDVRYPKRLTEAQKYDLRRVLGGNS* |
| evm.model.group6.964 | MAAEEDKSSDFYAVLGLKKECTATELRNSYKKLALKWHPDRFSASGNSKYVEEAKKKFQAIQQAYSVLSDANKRFLYDVGVYDCNDDDDENGMGDFLNEMATMMSQTKSNENRNETFEELQELFDDMFPSDIEAFGSSSRSTTPPTCSSSSPGSCNETYSISNKRNSREMSSSNLRDEPAQFEGFCVGTGGSSGRQQGCESSRRKNSRKGRR* |
| evm.model.group6.1530 | MAAAVGCGSASGSWARFGCSSGRNVRNDNKKGFRVSCVSSAVSDPYKILRIHPGAPESEVKKAFRQLALQYHPDVCKGSNCSVQFHQINEAYDVSCFKINFSYPSSLMFYN* |
| evm.model.group6.237 | MAMASWKSMLQITYSEEIVDGEPIYFSSNSLPIKSSNLEPAGHAFNAAALRLVGHCVEADTDEDDEKQVPDGKEHIYVQSSDSYRSKGKKKPGGEAKEQDHYALLGLSHLRYLATEDQIRKSYREAALRHHPDKQAALLLAEETEAAKQAKKDEIESHFKAIQEAYEVLIDPVRRRIYDSTDEFDDEIPYDCAPQDFFKVFGPAFLRNGRWSVNQTIPTLGDDKTPLKEVDNFYNFWYSFKSWREFPHADEFDLEQAETRDHRRWMERQNAKLSEKARKEEYARIRTLVDNAYKRDPRILRRKEEEKAEKQRKKEAKLLAKKLQEEEAARIAEEERQRKAEEDKKAAEAALNQKKLKEKEKKLLRKERTRLRSLSTPILSQHLLDLNDDDVESLCSSLDKEQLKSLCDLMERKEVLERAKILRERLEFDHKLKDEKDIRQNGSVAVNGRVPYSSNEKKEKPWGKEEIELLRKGMHKYPKGTSRRWEVISEYIGTGRSVEEILKATKTVLLQKPDSAKAFDSFLEKRKPAQAIASPLSTREEIKGLSNNSVADRRSSGSDNVPESSNQSGNSLNSDESTAANGSTLSTDQDSWSVAQERALVQALKTFPKETTQRWERVAAAVPGKTMGQCKKKFALMKESFRNNTAADKNTSSNTSNTDDSQGSSSPNEKSKTSADSEAENGTPLSSDVDIWSEVQEKALIQALKTFPKDTNQRWERVAAAVPGKTVNQCKKKFSSLKEDFRNKKNAV* |
| evm.model.group6.83 | MEYNKDEALRAKELAEKLLESNILGAKKFALKAQNLFPNLEGLSQFLEMIDVYVASEKKINGEPDYYAIFGVEPFVIEEVLKKKYKRMALSFHPDKNKSFGADGAFKIISEAWSVLSDKDKRSAYDLTLNIRAPNQAGSFSSVNLQETPRAPFHQHRTPARTQPTRSPCPSRLKTFWTSCNRCKVQFEYDKIYVNQNLLCGCCKKPFLAIEVSAPSVNGHSSRPWPFPCPQKDISIFPSGSRSVPDLGKRTSQAASYQASSKGVKRRNDGPSRNNEAPAKSSIKVPEKVEAASVSAPSAVSSGLNREKSAKKRRRDSLKCDSVKELSQVEIRSILKEKARKEIISQLNESVTEKSLSSDMKRAQTKDYVNDLKNGSINCPRTNQRRDNLYSDPKNKSQPHKSSAGDESDDIEANHDETVSMVVPDANFHNFDRDRVEESFVENQVWAAYDDDDGMPRYYALVHNIISRKPFKIQISWLNSKSSSEFGPLDWIGSGFTKTTGDFRVGKIVVNGKLNSFSHPVKCKKGARGVIQIFPTKGDVWALYRNWSPDWGELTADETVHKYDLVLVLEDYNEETGVLVVPLVKVAGFTSVFNQVSDKRDIQKIPREEMFRFSHQVPLYMLNGMEAENALKGCYELDPAALPLELLNVITGAEAAEDRVSEQPVMHSRTEGEGQAKAQLTSTGKTNATEEGGVAENAKHIITYVRRKKGRKTEGDADQSQK* |
| evm.model.group4.1253 | MAVDKEYYDVLGVEVDASHAEINKAYYKKARVVHPDKNHGDPEAAAQDFQVLGEAYQVLSDPETREAYDRCGKEEVIKDSMVDPTVLFGMMFGNEIFDNYVGQLALLSSPPPEFDPDLPPEVQKPKLEKIMKAVQEEREEKLVKNLKSRVELYVQGQKDEFLESAKVEARRLSQAAFGKAMLHTIGYIYTRQAAKEIGKGKRYMKVPFLAEWVRDKRHSRSTQSTAARAAIACIQLREEWNKCNEEENKDESTMKMAKEIKDKIFYSFWQMNVADIEKTVSRACQTVLKDPSASKDVLRLRAHGLKKLGKIFQGANGKKKQLVARSIDSMVDINSRSTS* |
| evm.model.group4.708 | MVRFHGLRLARRSLSSFLLHDSHSCLGDSLLKGGCESFSAVQFNQSRGLFYSNFGNVGSEKYWLKSGIFNDNIGAARSIHGTAQLSRDFYDVLGISKNATASEIKKAYYGLAKKLHPDTNKNDPEAEKKFQEVQKAYEVLKDEEKRQQYDQLGHEAFERAGNGEGPGFDPFGAGFNPFQDIFRNADIFNIFNREMGGEDVKVSVELSFMEAVQGCTKTLSILTDLTCETCGGTGVPPGTRPETCKRCRGSGMIVSQNGPFTLQSTCPNCGGAGKIVSSFCKSCKGRRVVRGPKTVKLNVMAGVDNNETIKIPRVGGADPDGSQPGDLYVMIKVREDPVFRREGADIHVDAVLSITQAILGGTIQVPTLSGDVVVKVSPGSQPGQKVVLRKKGIKVRNSFTFGDQYVHFNVSIPTNLTQRQRQLIEEFAKEEQREDDKGAAAGASG* |
| evm.model.group4.1870 | MGDPSKSQTPNFYGILGIPKSASLSDICKAYKFLVMKWHPDRNPSNKVEAEAKFSSINEAYRALSIKKREEANACNEDHEQKTHQKKKSTDYEKDKGSNGDGQELKISSPKLLSRTTSQISPLPSPTFSSSLSRRSTTPPPMDFYASFPKTSSGGNTPTTPSTPKEPTSLSTVASRRCTTPIFFSQSVVRRKPPPVEKKLECTLEELCHGCVKKIKITRDAISSSGLIVQEEEILMIKVKPGWKKGTKITFEGKGDERPGTLPADIIFSVDEKRHPLFKREGDDLELGVEVPLVQALTGCTVPVPLLGGGQITLSIDDIIYPGYEKIIPGQGMPTSKDGKRGDLKLKFLVEFPTELSNDQRSEVVSILQECS* |
| evm.model.scaffold245.29 | MGVDYYNILKVSRNATEEDVKKSYKRLAMKWHPDKNAVNTKEAEAKFKQISEAYDVLSDPQKRQIYDSYGEEGLKSGYYRSMERDNRGGRGYKFSPRDAEDIFEEFFGGFAVDKGKTAGVGGGGGGGGGGRLKKAAAMETKLPCSLEELYKGSRRKMKISRVVLDDSGKPGTVEEVLAIQIKPGWKKGTKITFPEKGNHEPGAAPGDLIFVVDEKPHPVFKRDGNDLILNQRISLLDALTGKNLTITTLDGRELPLTISNIVKPGHEMVIENEGMPISKEPGKNGNLRIKFDIKFPSRLTAEQKSDLRRVLGRTTE* |
| evm.model.scaffold452.41 | MDVETDHYAVLGLPSGEEGSRLSEQEINKAYRSKARELHPDKRRDDPNAHADFLKLQTSYEVLKDEKTRKLFDDLLRVKRERLHRQGKQDSKRRKMMSDLEERERSAFAVDPNEKAREEEVRIARKLKDEIARIRAMHTNKMSAATKPFKQDSTAQGKGSVDGGGTGLDKEKVLKVSWEKIGEDYTAQRLREIFEKFGSVEDVVIKSSKKKGSALVVMASKDAAVAATGNVLGDLSNPLLVLPLQPASSSVIFSAEKNESDGPKLSNLVGAGYQAFEDSVLEKMKKAAQSQK* |
| evm.model.scaffold259.19 | MCSYGVGQHLPRSDPLRISFSYSTGTYNHPIQFVSFRARPRRVPAIRSSLNDTVFERSTISFYELLGIPETGTLIEIKQAYKQLARKYHPDVSPPDRVHEYTQRFIQVQEAYETLSDPRRRELYDRDIANGVHLRLYCPKA* |
| evm.model.scaffold259.30 | MPRNQKTRTLMNPPMKFLPIPPKVGLAAGFRQLGHPHPPHRAHRLPTLRSRSPVVREIKIKKDYYEILGLEKSCSAEDVRKAYRKLSLKVHPDKNNAPGAEEAFKMVSKAFQCLSNEESRRKYDLVGSEEPVYERRGRGGGVNGMRGFNGFYDADVDADEIFRNFFFGGMHPATTGNFGGFAFGPGVRVRTGGVDQGSNWLRTLIQLLPVILILLVNFLPTSEPTYSLSRSYSYDYRLTTQKGVNFYVKSAKFEEQYPPNSPERVTIEQRVNDDYHRVLVHNCRLEWQQLHWGYRRETPNCDALKRFEAMVQ* |
| evm.model.scaffold201.1 | MNEENGEPNDRELYALLHVSPEASDEEIRRAYRQWAQVYHPDKYQSPQMKEIATQNFQRVCEAYEILTDENKRMIYDIYGMEGLTSGLELGPKLDKVEEIKEELERLRRQGEQEKVLAHIRSSGSILANLSWPDFLDGDGVMRGMAMASEVQSQISKRTSLSIGGNLAVDANSGGGAATAILSHHVSPVSTVEFMGSIGLQSLIGMQTSRQLSVHSTAIMGLTMSLRDGSVNLSNTWSRQLSETTTGNIQLSLGPESSIGVGWRKKEQKMSASGEIKIGTSSFGATAHCTHFFSSKSHGRIAGTVGSRALAIEIGGGRKISKFSTVRMLYSIGIQGLVWKFELHRGGQKLVVPILLSNHLNPVFATGAFIIPVSIYFTLKNFVLKPYYLRREKQKTLENIEKTQSQVQEARTAAKKAQQLLQNVANRKKTKQQEMNGLVVLKAVYGNQKALSNRHISEEREDEVVSQIMDVTIPLNFLVNDSGNLKLHQGVKKSGIMGFCDPCPGEPKQLYVEYTYRGARYEVTVDDYDELLIPQ* |
| evm.model.group12.31 | MVKETEFYDILGVSPSASDEEIRKAYYPKARQVHPDKNPNDPLAAEKFQVLGEAYQVLSDPVQRDTYDRNGKKCISKETMLDPTAVFALLFGSELFEDYIGHLAVASMSSTELSGESDSPDKVHHRFKAVQREREEKIARKLKDFLHHYVRGDTEGFIKQAKSEAHRLSHAAFGAEILHTIGYIYTRQAALELGKKAIYLGVPFMAEWVRNKGHFWKSQFTAAKGAFQLLQLQENIRNQSKMDGCASENDVKPLLRLNKETLMNSLWKLNVVDVEVTLLHVCQMVLQESNVKREELKTRAVALKILGKIFQGQKSRQNAGPSGKKLASAESRRKQFR* |
| evm.model.group12.625 | MSNLRVICRPYSVFSSMACCYRSMVRFSPIKNDSNNFRLGSSSSSSSVFAVWFDKLGAVKYEPRVRLNQQRRMVATRASNWTDSKSPYETLELERDADEEQIKVAYRRLAKFYHPDVYDGRGTLEEGETAEARFIKIQAAYELLIDVEKRRKYDNDNRVNPMKASQAWMDWLMKKRKAFDQRGDMAVAAWAEQQQRELNLRARRLARSKIDPEEERKILVKEKKASEENFHNTLRRHTLVLRKRDLMRKKAEEDKKKIIGQLLAAEGLELEKDEEEQ* |
| evm.model.group12.864 | MLRSQSSWKDRRSFRTSFQEEAWKRYNRRMQEEYEDEMERVERIRRMQSVFNRERNKFKRGYESWQDNGAGGYNQHFQRNDWYWKADTSFRDHNSNFRETPRASASAPLSHHYSVLGLDRLRTKPYTDDEIKSAFRTKAKEFHPDQNKDNKEFSEAKFKEVMVSYEAIRSERKNNGHQ* |
| evm.model.group12.627 | MSNLRVICRPYSVFSSMACCYRSMVRFSPIKNDSNNFRLGSSSSSSSVFAVWFDKLGAVKYEPRVRLNQQRRMVATRVSNWTDSKSPYETLELERDADEEQIKVAYRRLAKFYHPDVYDGRGTLEEGETAEARFIKIQAAYELLIDVEKRRKYDNDNRVNPMKASQAWMDWLMKKRKAFDQRGDMAVAAWAEQQQRELNLRARRLARSKIDPEEERKILVKEKKASEENFHNTLRRHTLVLRKRDLMRKKAEEDKKKIIGQLLAAEGLELEKDEEEQ* |
| evm.model.group12.742 | MACVFSPNQFFKNFCVGRQLLYNRPVESYAGKVPQFQRPPIGTVLRENMLALHLMKLLSSKRFVHATGAFRSTERDYYEILGLSEDATRDEIKKAFHALAKKYHPDANKNNPSAKRKFQEIRDAYETLQDPEKRAQYDRMRESSARAKNAEYPNRDWDDLRYAHRTQFSNTFQKIFSEIFESESENLAGDIQVELVLTFSEAAKGCTKHLSFDADVPCDSCYGRGHPPDAERKPCPTCQGIGRVTIPPFTTTCSTCKGFGRIIKEYCMACKGSGVCPGVKDVKVTIPAGVDSGDTIRVPKAGNSGGWGRSPGNLFIKLKVSEDSIFSRQGADVYVDSDISFTQAILGGKVEVPTLSGKMQLQIPRGVQHGQLVLLRGKGLPKSGYFVDHGDQYVRFCIKFPSTVTEKQRAILEEFEKEIIDQNSTFAEDSWWQYWVERAIGPKFILELSILMLIILFLGKVLN* |
| evm.model.group12.424 | MASTSFPLSNQITGRKFSASSPPLRRTQRISASSPPLRRTQRISAAYATAEGVPVIGSENTSQTVSLYEVLGIQTAATSQEIKFAYRRLARVLHPDAAVGGDASSADKFMRVHAAYITLSDLEKRAVYDSSLFWRRRRTAILTASGFSEVTMRRRTWETDQCW* |
| evm.model.group7.3496 | MELGMKLCGFDLVAWRGFIYAIFILNFVFFCQLILLQPLVAAIDGKPSDSAALFERVSQSVKVKKYSEAIAELNAAIEADPAFSEAYWHRASLLRQLCRYEESEESYKKFLKMKPGNSAAEKELSQLHQAQNALDSANNLFDSGDFTKALEYIEKVVLVFSPACSKAKLFKARLLIATKDYSSAISEAGFILKEDEDNLEALLLRGRAYYYLADHDVAIRHYQKGLRLDPEHGELKKAYFGLKNLLKKTKSAEDNASKGKLRVAVEEYRAALAFDPNHSAHNVHLHLGLCKVLVKLGRGKDAVTSCTEVLEIDGDLVEALVQRGEAKLLVEDWEGAVADLKSAAEKTPQDMNIREALMRAERSLKLSQRKDWYKILGVSKTSSMSEIKKAYKKLALQWHP* |
| evm.model.group7.1886 | MAGSKMEGPSAPALRRDPYEVLCVSRDSSDQEIKTAYRKLALKYHPDKNANNPEAAELFKEVAYSYSILSDPEKRRHYDSSGFEALDAEGMDMEIDLSNLGTVNTMFAALFSKLGVPIKTTISANVLEEALNGTVTVRPLPIGTSVSGKVEKQCAHFFGVTISEEQAELGIVVRVTSAAQSKFKLLYFEQDANGGYGLALQEDSEKAGKVTSAGMYFLHFQVYRMDSTVNALAMAKDPEAAFFKRLEGLQPCEVSELKAGTHIFAVYGDNFFKPANYVIEALCAKSYEDTTEKLKDIEAQILRKRNELRQFETEYRKALARFQEVTNRYSQEKQSVDELLKQRDIIHSSFTVARSVSLASGSGHFSNGSSSKVPSEDYKSESPGEEGSSDSKDKSSKKKWFNLNLKGSEKK* |
| evm.model.group7.2700 | MGSHPRKSRSRKPSREPSTSSAATSSSDDELESTSKYRKRRRDRDRDREEMRRSKKSRRDNERSKKKKKHSRRDREKHKRKERKKRRYDLDNESGSGSGSDSELESPEDLVKYVLKEFPAVAGDLEQLLQMIDEGQAVDIRGLSEKSLITHLRRLFISLNLKENGDKVFLLPQKASPTLDVVGPIIRSHVQLEKEHDPLDSQRDDHSVPPNTEHKDVTGNVDDSKEDSVGPRRRVIGPEMPSAELLAAAAKLTEAEAELRDAELDGDTDLFIGPPPPAVVTEAESANEAERFEEITRIIGAEDSSPYDILGVNKNMSDQNMKKRYWKLSLMVHPDKCSHPQAQQAFVKLNKAFKDLQDPDKRKALDDKIKEKEDKEAFQVELKAMREAAQWRRLQGISMEGDDILLAEMDAKAAPKREEWMTTLPPERKPGMSMHSTTSFSKSSKEGRGDTSAWTDTPSDRAQKAKINYLEAYNEAAALASNEQEKKRANKDAELVDQYNKAKRSKTLVEKHQEMRKSGTKAKSKEKVKDDWEGNHPWKPWDREKDLTAGRQSVNFDTENMAAGLSSRFSSGSFQRNFL* |
| evm.model.group7.3245.1 | MMQAYGYLVLGPTSFDGAGCSKWIASSYWSTSRGRHRFTASAAINGHRNHYDVLGVSPNASSSDIKKAYRLRALKYHPDVNKEVGAIEDFKSIRLAYDVLINETTRSEYDRAQCHESTRSPLRDEWATNPEWEDGLRFYRWANLRRKMRYERYWEQYHAREEQFPFYDDVDEVSEEENQDEERGSFMEVLRSTFISLFLMQTVGIRLSLTYSGLMALIDRKLDAGYKLGYLFAWILGGRTGILLTMFLSFASWVCGKTSSSIVALVVVAMWFGTNLARYAPIPQGALLTLLYMSMKLQVDLT* |
| evm.model.group7.3671 | MSFCKISSSSSRAPFHFFPNSNQPNRTLITEDISFKFQVSKSRISIRNPSGPARRARTTARTRAFYAPTAGTEESLYELLGVSESGSTLSDIKKAYKKMARKYHPDVSPPDRVNENTKKFIMVKEAYETLSNPQTRALYDRDLANGSGFRFSARSSYQYNDQRVAETGEWKQRWQAQVDELKRRSMNPSSGRMSWGARMRAQR* |
| evm.model.group7.1828 | MEYGTGSPSYYGVLGVSLCSSDEEIRRAYRKLAMQWHPDKWTRSPALLGEAKQKFQQIQEAYSVLSDRGKRTLYDAGLYNPDEEEEDEVEGFADFVQEMMSLVNDARKEGKNYSVEELQRMFWDMAQGFEIPEWSNCSSLQQPMYESQCSLGPFKLYESTNSCEVNMIQGNQNLSKPGFEAYVARHFF* |
| evm.model.group7.1264 | MDGKYKTTYKDYYKVLEVDYDATDEKIRLNYRKLALKWHPDKHKGDSAVTAKFQEINEAYTVLSDPDKRLEYDLNGNYEIDKYTLREYLSRFKGMILTCNGLGISHTSIWSQPLMELHDLKEK* |
| evm.model.group7.1073 | MECNRDEALRAKAIAEGKIEKRDFAGAKKFALKAQNLYPGLDGVSQMLTTLDVYVSAENKISGEIDWYGVLGVNPSADDETMKKQYRKLVLMLHPDKNKSVGADGAFKLVSEAWSLLSDKAKRLAYNQRRGFRGVHQKVQMHPTGGPSASSRANGIFNHVSRTTSVPKPQNNSATSVPKPPPPTPPPVYQRTDTFWTICHRCKMHYEYLKVYLNQTLLCPHCKEAFMASETSPPFNFSKSQNQVPKQRQHTPTDRTPSRNAHVPARNVPTAQNSGPGQASANSFRYHPYQRNPHSGSVSAASTDPSIAAKAANVVQQAQDKLKRAYTESHASAGWEGSVKKSKLDDGRSQCGMKYNMSQGNFGFGTASGSASGSRIYGFSAPYRQPNSTRDLTPLETRSMLTRLAVKEILDYLKAKTTVKDEDKEKDITKESKNEKRRSTTDVEGHDQNGNSDLSDQPGKGQLSHPADNESKEKSAAALMSVPDPDFHDFDEDRAESSFGDNEVWAAYDDDDGMPRFYALVNKVLSRKPFKLRISWLNSKTNTEFSTIDWVGSGFYKTCGEFRVGRYEACKSINSFSQKVNWSKGPRGSILILPQKGDVWALYKNWSSDWNQHTPDEVIHKYDMVTVLDDYNEEQGVRVAPLVKVVGFKTVFRPNLDPKLIKKIPKEEMFRFSHRVPYHLLTGAEAQNAPKGCLELDPAATPLELLQVITEGSEVPTVPNAAQAVNDKEVS* |
| evm.model.group7.2771 | MAIIPCGSTWATRWGTQPQFVLRPSITNKVSNFPHCTSSRTGSLASQNSNFLSQESLNVLFHSASYNNPRQRRGVRLIVRADKDYYSVLGVSRNASKSEIKSAYRKLARSYHPDVNKEPGAEEKFKELSNAYEVLSDDEKRSIYDKYGEAGLKGSGMGMGDFSNPFDLFESLFEGIGGMGGMGGMGGRGSRNRATEGEDQVYNLILNFKEAVFGVEKEIEIARLETCSTCDGSGAKPGTKASKCSTCGGQGQVVSSARTPLGVFQQVMTCSTCGGSGEISTPCNTCSGDGRVRKSKRISLKVPAGVDSGSRLRVRSEGNAGRRGGPPGDLFVIIDVLPDPVLKRDDTNILYTCKVSYIDAILGTTMKVPTVDGPVDLKIPAGTQPSTTLVMAKKGVPLLNKSNMRGDQLVKVQVEIPKRISSEEKKLIEELANLNKAKAPNSRR* |
| evm.model.group7.996 | MADSEENNALFPIFILSMIALPLVPYTVLKLFHAASKKTKTIHCGCSDCARSGKYRKSVFRRIANVSTFGNFTLVLLWIIMGILVYYIKNISREIQVFEPFSILGLGPGASDSEIKKAYRRLSIQYHPDKNPDPAAHKYFVESISKAYQALTDPISRENFEKYGHPDGRQGFQMGIALPQFLLNIDGASGGIILIWIVGVCILLPLVMAVVYLSRSSKYTGNYVKNDTLGAYFHLMKPSLAPSKVMEVFIKAAEFLEIPVRRSDDEPLQKLFMTVRSELNLDLKNIRKEQAKFWKQHPALVKTELLIQAHLTRETAALSPDLERDCKRMLELSPRLLEELIKMAIIPRSSKGHGWLRPAIGVIELSQCIVQAVPLSARKAAGGSADGVAPFLQLPHFNDDIIKKIARKKVRTFQEFQGMSIQERADLLSQAAELSPPQLQDVEKVMEMIPSLTVEVTCETEGEEAIQDGDVVTVQAWVTLKRANGLIGALPHAPHYPFHKEENFWFLLADPNSNSVWFSQKINFMDEVAAISAASTTIDEKMEVLGVPPKEKSAAIKEAVEKVKSGSRLAMGKFLAMAEGNYNLSCYLLCDSWIGCDQKSTLKLKVMKRTRAGTRGGLVNDGGLVVEEGFEEEEEVEEEEEEEEDEDIESEYSEDEDDKLKANKKGTANGKGNGKGKQSSSEISGSNED* |
| evm.model.group7.3737 | MFGRSHKKSDNSRYYEVLGVPKTASPDDLKKAYKKAAIKNHPDKGGDPEKFKELAHAYEVLSDPEKREIYDQYGEDALKEGMGGGGGMHDPFDIFSSFFGGGPFGGGGSSRGRRQRRGEDVVHPLKVSLEDLYVGTTKKLSLSRNVLCSKCKGKGSKSGASVKCSGCQGTGMKVTIRQLGPGMIQQMQHACNECKGTGETISDKDRCTQCKGEKVVQEKKVLEVHVEKGMQNGQKITFPGEADEAPDTVTGDIVFVLQQKEHPKFKRKGDDLFVEHSLLLTEALCGFQFILSHLDGRQLLIKSEPGEVVKPNSYKAINDEGMPMYGRPIMKGKLYIHFNVEFPDSLGPDQVAALEKILPPKPQSQLTEMEIDECEETTLHDVNMEEEMRRKAQYQEAYDEDDEDMHGGAQRVQCAQQ* |
| evm.model.group7.3669 | MISSISFNSHATRSKIPIKTPSGSCRTRSTTRIQALYAPAESKETLYELLGITEPENATLSNIKKAYKQMARKYHPDVAPPDLVEEYTSRFMMVHEAYETLSDPQTRALYDTDLVAGFGRKSGEYRRKMAQQREWKMSWEAQVEGQKLRTRDPNSWAARVRRANYGC* |
| evm.model.group7.706 | MAALPLTSSDSLYTSTKMSHNDGKAGSVDVVWPEEKALRLKTLAEQKYTSSNLKSALKYAKRAHKLHPSLEGLQGLLTALKIIHTSTIPIFESSSFTQNSAPDYYKILQVERFSHINTIKKQYKKLALILHPDKNPFVASEEAFKLVGEAVGVLLDKVKRKEYDMRLRIVMQSKATEEIELGAEAEAKTFWTACSTCRLLHQFERKYMGHNLMCPRCKKSFKATEVEEERSNAGDLNKEEKNERVGAGQKRVSARIREKIAKGGNLGILDKVSLGVKRKTSTVGEVLKRSRVKVARNMDGDGDSESRDIEVVNELKSRRDEVEDAAKSGGVEVVESERTKRAKVREEESMTLSQMQMLVKKKKLVEGQLTLKEKEVNAEKLKLKENGGEREKEKGRERDNEEEEEEREEEEEREVEERVEEDKEKEKVNVREKMNRRRVSKDRNLEVEKIMSKFPVHSEIERDRASKKDNMEVMPVEKEKEKEMEDTREKRQRRRVSKYGNWEVMKTETSKNHVESEIKKRIATKKGKLEIMPVEDSDFHDFDKDRKEKSFKKGQVWAVYDDVDGMPRQYALIDEILSVNPFEVALIWLQFQSNGDEELTSWHRKGFHISCGSFKVSGKVTIKYLNLFSHVVNCERAAREMYRIYPKKGSVWALYNSDALDAEERTQKDKDRQCYDVVVSLSSYSDIYGLSIAHLEKVDGFRTVFKRREIGANAVTCLGKSDLKLFSHQIPAKKLSVEEASGLPKDCWELDPASLAPHMLIAS* |
| evm.model.group7.2348 | MHSSATLLLHTPSAIPNLKTTSLSTINHPRCAKPFFSPQKKTRNYDNFPACRASQYSPITEFNLYELLGVDASSDRAQIKEAYRSLQKRCHPDIAGPAGHDMAIVLNETYALLSDPDFRLAYDKELAKVADLQGYTGRPMYSAWLGSESEQRAVFVDEVKCVGCLKCALFADKTFAVESIYGRARVVAQWADPEHKIQQAIASCPVDCISIVERSNLAALEFIMSKQPRGSVRIGAGNTVGARTTDIFDETEKFQARYAKHQASTDKSKESEARMASRISAIQAIQMISNWLYWQSPSADGSPLATQNSQPSSRKHHGPSFKKLKAAAEARKQSTATSGRKELLSNMNEYWVPSALVLPEGTSPGSGFKPESKPRVDLSPTQTTNQPRNDEFLSPKEDSGNPIRWGVPMGTAFVAAAVVRLQLGGEPVGGLKDHIGGSMVLAIVNSSWLQVVLAGVTWYLIGMYVVELVYILKRK* |
| evm.model.group7.2512 | MLSDEAKILLGFPPNSLPSPSQVKAAYRRKAWETHPDRFPAHEKAQAECNFKLISEAYTFLNSGGSRHDVPREASYSRVVKTGVPRAHGGRRNLRVASVPFLLIIFGALTLGGSTASRAYRRQKEAHPTFNPFLP* |
| evm.model.group7.291 | MILATRLTAKVDENADINGIRKQYHKLALQLHPDKNKHSKAEAAFKLVSEAYYCLSDNARRAAFDSQKTNSCNKCNTVIPSTNKNPPNYSNIKIQEPYQEKSRSIRTQKSKKELQTKLKEQANIIERCLIINARLSRKESLNANETEKEHPVFNPSDYQHKGYPHFRTINHKKIEELRAALLIRNRCTNSSPIFEYRPERTPFMSRCAGTRDH* |
| evm.model.group7.544 | MGVDYYNILKVGRGATEDDLKKAYRRLAMKWHPDKNPNNKNEAEAKFKQISEAYEVLSDPQKRQIYDQYGEEGLKDMPPPGSTGSQNGFNPRNAEDIFAEFFGSSPFGFGSSGAGRSMRFSSDGGGMFGGVSGADNMFRNSSSDGSGVNMPKKPPPVEIKLPCSLEELYTGSTRKMKISRQVFDSNGRLVTETEILTIDVKPGWKKGTKITFQDKGNEQPNQLPADLVFVIDEKPHNIFKRDGNDLMMSYQVTLAEAIGGTTVDVTTLDNRNLSIPVNDIINPGYELVVSREGMAITKEPGNRGDLKIRFEVKFPTRLTPEQRVALKRALGG* |
| evm.model.group7.3013 | MDHYKVLGVHKNASKEEIKQAFRKLAMEFHPDKHSQSSNHLRDSATHKFKQLSEAYETLIDDRKRADYNLKRNAYGPQSRRYDYNPNRGGGRGTYSYSNSYGYGYGYGYGGSARRSGSDGIFTRFETCLRFLTTRSFLLNATFVGLLLGGMFVVDAGGKALWKMRNPGKSFEEAMESVEKAKAPKDSS* |
| evm.model.group7.3670 | MAKKYHPKAAPRKKVGKCTKRFLRVHEAYQTLSDPQKRAVYDKDNQKIAQQREWKMRCRTQLEGLKLRATARDPNSWAAVVRTETALGLLISGGKIVFSDN* |
| evm.model.group7.4151.1 | MHGFAYSLTSPSILRLNPFPFPSLKSSSSALVCRIQYGHNVERFVCTAEAFLHSYTRDYQCRKKKKRGNFRGTQIRASGRESPYEVLGVSPSATTKEIKRAYRKLALKYHPDVNKEAGAQEKFMRIKHAYNTLLNSRSRGKYDAGNQGSDFSYSANGRYQTKTREAEEEFYGLGDILRDAQKTIEDFFRDLQEEFRNWEAAVATQGKPKSLWEELAEIGEDLVEFLEKELNITDEDEKNDERSSKGEASSGTKETASADGEEADKGSSIDENIDEIEAALAQLKKELGL* |

### Supplementary Table 5.

Information of DnaJ gene family in *Catalpa bungei*.

| **Gene ID** | **Sequence ID** | **Type** | **MW**  **(kDa)** | **PI** | **Chromos-ome** | **location** | **Subcellar Location** |
| --- | --- | --- | --- | --- | --- | --- | --- |
| *CbuDnaJ01* | evm.model.group0.1338 | C | 5.28 | 37.33 | group0 | 53447339-53453753(+) | N |
| *CbuDnaJ02* | evm.model.group0.1822 | C | 5.03 | 18.62 | group0 | 58408092-58410003(+) | N |
| *CbuDnaJ03* | evm.model.group0.1914 | C | 4.92 | 15.85 | group0 | 59040827-59044003(-) | N |
| *CbuDnaJ04* | evm.model.group0.2150 | C | 6.04 | 152.70 | group0 | 61481514-61493049(-) | Chl |
| *CbuDnaJ05* | evm.model.group0.2332 | C | 9.35 | 30.56 | group0 | 65696618-65701570(+) | Cyt |
| *CbuDnaJ06* | evm.model.group0.2339 | A | 6 | 46.33 | group0 | 65781283-65785530(+) | N |
| *CbuDnaJ07* | evm.model.group1.1390 | C | 7.2 | 82.70 | group1 | 19215178-19217409(+) | N |
| *CbuDnaJ08* | evm.model.group1.2318 | C | 7.43 | 60.75 | group1 | 44210929-44217790(+) | P |
| *CbuDnaJ09* | evm.model.group1.471 | C | 9.23 | 35.52 | group1 | 9536338-9542926(-) | V |
| *CbuDnaJ10* | evm.model.group2.1016 | C | 6.72 | 143.26 | group2 | 23473931-23480551(+) | N |
| *CbuDnaJ11* | evm.model.group2.1094 | C | 6.31 | 32.91 | group2 | 24157728-24161341(-) | N |
| *CbuDnaJ12* | evm.model.group2.1135 | C | 9.12 | 30.94 | group2 | 24428786-24431986(-) | N |
| *CbuDnaJ13* | evm.model.group2.1334 | C | 9.43 | 62.88 | group2 | 25895224-25899693(-) | V |
| *CbuDnaJ14* | evm.model.group2.1856 | C | 5.48 | 76.96 | group2 | 33023453-33031405(+) | P |
| *CbuDnaJ15* | evm.model.group2.203 | C | 5.81 | 280.67 | group2 | 11107564-11129070(-) | P |
| *CbuDnaJ16* | evm.model.group2.346 | C | 6.89 | 58.96 | group2 | 14549682-14556082(+) | N |
| *CbuDnaJ17* | evm.model.group2.349 | C | 6.47 | 91.45 | group2 | 14625642-14628198(+) | N |
| *CbuDnaJ18* | evm.model.group3.1209 | C | 8.87 | 30.80 | group3 | 21954325-21956352(-) | N |
| *CbuDnaJ19* | evm.model.group3.897 | C | 9.53 | 17.77 | group3 | 19769128-19769586(+) | N |
| *CbuDnaJ20* | evm.model.group4.1253 | C | 8.41 | 38.55 | group4 | 24755256-24763108(-) | N |
| *CbunaJ21* | evm.model.group4.1870 | B | 8.82 | 40.95 | group4 | 35942131-35943571(-) | N |
| *CbuDnaJ22* | evm.model.group4.708 | A | 8.64 | 48.50 | group4 | 19646992-19654843(-) | M |
| *CbuDnaJ23* | evm.model.group5.1476 | C | 6.43 | 36.33 | group5 | 39723669-39727087(+) | Cyt |
| *CbuDnaJ24* | evm.model.group5.1544 | A | 9.4 | 47.61 | group5 | 40092311-40097878(-) | Chl |
| *CbuDnaJ25* | evm.model.group5.275 | C | 5.43 | 44.23 | group5 | 8088369-8097588(+) | Cyt |
| *CbuDnaJ26* | evm.model.group5.504 | A | 5.86 | 46.66 | group5 | 16200776-16203265(-) | N |
| *CbuDnaJ27* | evm.model.group5.587 | C | 9.04 | 35.99 | group5 | 17318629-17319588(+) | N |
| *CbuDnaJ28* | evm.model.group5.913 | C | 9.15 | 37.14 | group5 | 30974567-30975565(-) | N |
| *CbuDnaJ29* | evm.model.group5.968.1 | C | 9.56 | 28.57 | group5 | 32699940-32705519+ | Chl |
| *CbuDnaJ30* | evm.model.group6.1530 | C | 9.15 | 12.18 | group6 | 27199283-27202447(-) | Chl |
| *CbuDnaJ31* | evm.model.group6.237 | C | 8.16 | 85.38 | group6 | 8498781-8501708(+) | N |
| *CbuDnaJ32* | evm.model.group6.489 | B | 9.08 | 38.86 | group6 | 13494793-13498547(-) | Cyt |
| *CbuDnaJ33* | evm.model.group6.83 | C | 8.84 | 80.89 | group6 | 3477150-3479535(+) | N |
| *CbuDnaJ34* | evm.model.group6.964 | C | 5.17 | 23.75 | group6 | 18229589-18233045(+) | N |
| *CbuDnaJ35* | evm.model.group7.1073 | C | 9.03 | 81.35 | group7 | 11463750-11469221(-) | N |
| *CbuDnaJ36* | evm.model.group7.1264 | C | 6.42 | 14.58 | group7 | 13337994-13340983(-) | Cyt |
| *CbuDnaJ37* | evm.model.group7.1828 | C | 4.64 | 21.71 | group7 | 18767727-18769676(-) | N |
| *CbuDnaJ38* | evm.model.group7.1886 | C | 5.94 | 45.60 | group7 | 19403963-19410013(+) | N |
| *CbuDnaJ39* | evm.model.group7.2348 | C | 9 | 52.16 | group7 | 23369232-23373227(+) | Chl |
| *CbuDnaJ40* | evm.model.group7.2512 | C | 10.38 | 14.99 | group7 | 24734925-24737839(-) | E.R. |
| *CbuDnaJ41* | evm.model.group7.2700 | C | 8.84 | 65.94 | group7 | 26165660-26170444(-) | N |
| *CbuDnaJ42* | evm.model.group7.2771 | A | 9.32 | 47.92 | group7 | 26897249-26902606(+) | Chl |
| *CbuDnaJ43* | evm.model.group7.291 | C | 9.69 | 24.70 | group7 | 2788500-2793434(+) | N |
| *CbuDnaJ44* | evm.model.group7.3013 | B | 9.66 | 21.27 | group7 | 29367747-29371351(-) | Chl |
| *CbuDnaJ45* | evm.model.group7.3245.1 | C | 7.02 | 34.76 | group7 | 31137227-31140117+ | Chl |
| *CbuDnaJ46* | evm.model.group7.3496 | C | 8.31 | 44.94 | group7 | 33316359-33321294(+) | E.R. |
| *CbuDnaJ47* | evm.model.group7.3669 | C | 9.57 | 19.19 | group7 | 34650183-34651107(+) | M |
| *CbuDnaJ48* | evm.model.group7.3670 | C | 10.33 | 11.60 | group7 | 34653740-34654515(+) | Chl |
| *CbuDnaJ49* | evm.model.group7.3671 | C | 10.35 | 23.27 | group7 | 34656987-34657726(+) | N |
| *CbuDnaJ50* | evm.model.group7.3737 | A | 6.06 | 46.27 | group7 | 35106752-35110420(-) | N |
| *CbuDnaJ51* | evm.model.group7.4151.1 | C | 5.75 | 32.67 | group7 | 39121407-39123962+ | Chl |
| *CbuDnaJ52* | evm.model.group7.544 | C | 7.7 | 36.69 | group7 | 4578300-4580677(+) | Cyt |
| *CbuDnaJ53* | evm.model.group7.706 | C | 8.75 | 87.64 | group7 | 6232919-6235210(-) | N |
| *CbuDnaJ54* | evm.model.group7.996 | C | 5.73 | 76.74 | group7 | 10311889-10318340(-) | P |
| *CbuDnaJ55* | evm.model.group8.158 | C | 9.74 | 17.13 | group8 | 6437749-6438335(-) | Chl |
| *CbuDnaJ56* | evm.model.group8.197 | C | 7.95 | 60.32 | group8 | 8086199-8092487(-) | N |
| *CbuDnaJ57* | evm.model.group8.552 | B | 8.64 | 37.45 | group8 | 15512367-15516576(+) | Cyt |
| *CbuDnaJ58* | evm.model.group8.797 | C | 8.57 | 16.04 | group8 | 17933435-17934977(-) | Chl |
| *CbuDnaJ59* | evm.model.group10.186 | C | 4.9 | 45.56 | group10 | 1726378-1727960(-) | Chl |
| *CbuDnaJ60* | evm.model.group12.31 | C | 9.23 | 38.27 | group12 | 357257-362598(-) | Cyt |
| *CbuDnaJ61* | evm.model.group12.424 | C | 10.62 | 18.13 | group12 | 7738373-7738864(-) | N |
| *CbuDnaJ62* | evm.model.group12.625 | C | 9.47 | 32.62 | group12 | 22583642-22590354(-) | Chl |
| *CbuDnaJ63* | evm.model.group12.627 | C | 9.47 | 32.65 | group12 | 22610464-22616429(-) | Chl |
| *CbuDnaJ64* | evm.model.group12.742 | C | 8.73 | 52.14 | group12 | 25664007-25672905(-) | Chl |
| *CbuDnaJ65* | evm.model.group12.864 | C | 9.66 | 21.78 | group12 | 28980474-28985608(-) | N |
| *CbuDnaJ66* | evm.model.group13.333 | C | 5.78 | 68.58 | group13 | 13887770-13892769(+) | N |
| *CbuDnaJ67* | evm.model.group13.44 | C | 10.02 | 16.48 | group13 | 2648203-2649345(+) | Chl |
| *CbuDnaJ68* | evm.model.group13.704 | C | 5.55 | 25.22 | group13 | 18973380-18978232(+) | N |
| *CbuDnaJ69* | evm.model.group13.908 | C | 9.05 | 36.74 | group13 | 22041349-22046302(+) | Chl |
| *CbuDnaJ70* | evm.model.group13.964 | C | 7.65 | 17.45 | group13 | 23054744-23058317(-) | Chl |
| *CbuDnaJ71* | evm.model.group14.178 | C | 9.21 | 29.44 | group14 | 1370322-1371441(+) | E.R. |
| *CbuDnaJ72* | evm.model.group14.46 | C | 8.61 | 82.25 | group14 | 436948-442292(+) | P |
| *CbuDnaJ73* | evm.model.group15.557 | C | 4.89 | 20.56 | group15 | 18050833-18053773(+) | N |
| *CbuDnaJ74* | evm.model.group15.872 | C | 5.42 | 30.72 | group15 | 24501927-24503522(+) | Chl |
| *CbuDnaJ75* | evm.model.group16.670 | C | 5.34 | 18.34 | group16 | 20264143-20267038(+) | N |
| *CbuDnaJ76* | evm.model.group16.75 | C | 8.71 | 47.96 | group16 | 901912-909229(+) | M |
| *CbuDnaJ77* | evm.model.group16.800 | C | 5.25 | 55.78 | group16 | 21971818-21973341(-) | N |
| *CbuDnaJ78* | evm.model.group17.100 | C | 8.36 | 46.57 | group17 | 1501832-1507970(+) | N |
| *CbuDnaJ79* | evm.model.group17.397 | C | 8 | 86.32 | group17 | 7463171-7466900(-) | N |
| *CbuDnaJ80* | evm.model.scaffold201.1 | C | 8.41 | 59.45 | scaffold201 | 11635-22658(+) | N |
| *CbuDnaJ81* | evm.model.scaffold245.29 | B | 9.29 | 35.04 | scaffold245 | 283464-286437(-) | Cyt |
| *CbuDnaJ82* | evm.model.scaffold246.24 | C | 9.14 | 12.66 | scaffold246 | 126953-127516(+) | N |
| *CbuDnaJ83* | evm.model.scaffold259.19 | C | 9.35 | 16.46 | scaffold259 | 304006-307799(+) | Chl |
| *CbuDnaJ84* | evm.model.scaffold259.30 | C | 9.63 | 35.81 | scaffold259 | 433653-438578(+) | M |
| *CbuDnaJ85* | evm.model.scaffold291.46 | C | 4.7 | 20.65 | scaffold291 | 416794-418533(-) | N |
| *CbuDnaJ86* | evm.model.scaffold425.3 | B | 6.25 | 39.13 | scaffold425 | 7740-12021(+) | V |
| *CbuDnaJ87* | evm.model.scaffold452.41 | C | 9.17 | 32.60 | scaffold452 | 322573-329692(-) | N |
| *CbuDnaJ88* | evm.model.scaffold69.59 | A | 8.69 | 48.73 | scaffold69 | 540537-545172(+) | N |
